# Characterization of a novel HDAC/RXR/HtrA1 signaling axis as a novel target to overcome cisplatin resistance in human non-small cell lung cancer

**DOI:** 10.1186/s12943-020-01256-9

**Published:** 2020-09-02

**Authors:** Wenjing Wang, Mengyue Zhao, Lijuan Cui, Yong Ren, Jingyuan Zhang, Junli Chen, Lina Jia, Jiayu Zhang, Jingyu Yang, Guoliang Chen, Charles R. Ashby, Chunfu Wu, Zhe-Sheng Chen, Lihui Wang

**Affiliations:** 1 Department of Pharmacology, Shenyang Pharmaceutical University, Shenyang, PR China; 2 Benxi Institute of Pharmaceutical Research, Shenyang Pharmaceutical University, Shenyang, PR China; 3 Department of Pathology, Wuhan General Hospital of Chinese People’s Liberation Army, Wuhan, PR China; 4 Key Laboratory of Structure-Based Drugs Design and Discovery of Ministry of Education, Shenyang Pharmaceutical University, Shenyang, PR China; 5 Department of Pharmaceutical Sciences, College of Pharmacy and Health Sciences, St. John’s University, Newyork, NY 11439 USA

**Keywords:** Cisplatin resistance, Lung cancer, HtrA1, HDAC, RXR

## Abstract

**Background:**

Cisplatin is a first-line drug for the treatment of human non-small cell lung cancer (NSCLC); however, the majority of patients will develop drug resistance after treatment. In order to overcome cisplatin resistance, it is important to understand the mechanisms underlying the resistance.

**Methods:**

A gene microarray was used to screen for genes related to cisplatin resistance in NSCLC cell lines. Subsequently, the correlation between the HDAC, RXR and HtrA1 genes, in NSCLC, were verified using gene manipulation. Immunohistochemical staining was used to detect HDAC, RXR and HtrA1 expression in NSCLC specimens. Proliferation, migration and invasion assays were performed in vitro and in vivo to determine the role of the HDAC/RXR/HtrA1 signaling axis in cisplatin resistance, and luciferase reporter analysis and ChIP assays were performed to ascertain the mechanisms by which HDAC and RXR regulate the expression of HtrA1. Furthermore, in vitro and in vivo experiments were conducted in NSCLC cisplatin-resistant NSCLC to elucidate the effect of the low molecular weight compound, DW22, which targets the NSCLC cisplatin resistance HDAC/RXR/HtrA1 signaling pathway.

**Results:**

HtrA1 was identified as a cisplatin resistance-related gene in NSCLC cells. The regulation of HtrA1 by HDAC and RXR significantly decreased the efficacy of cisplatin in NSCLC cells resistant to cisplatin. Immunohistochemistry results showed a negative relationship between HDAC1 and HtrA1, and a positive relationship between RXRα and HtrA1 in NSCLC patients’ tissues. Notably, the expression of HDAC1 and HtrA1 can be considered as biomarkers for the efficacy of platinum-based drugs and prognosis in NSCLC patients. Mechanistically, the heterodimers of the nuclear receptor RXR, in combination with the enzyme, HDAC, regulate the transcription of HtrA1 in NSCLC cells. The rescue of HtrA1 expression by dual targeting of HDAC and RXR with the compound, DW22, significantly inhibited the proliferation, migration and invasion of NSCLC cells resistant to cisplatin, and induced NSCLC cell apoptosis.

**Conclusion:**

Our results indicate that HtrA1, a cisplatin resistance-related gene, is synergistically regulated by HDAC and RXR in NSCLC. Targeting the HDAC/RXR/HtrA1 signaling axis can rescue HtrA1 expression and reverse cisplatin resistance in NSCLC.

## Background

Lung cancer is one of the most common malignant tumors worldwide, and is associated with a high rate of morbidity and mortality [1]. Non-small cell lung cancer (NSCLC) accounts for about 85% of global cases of lung cancer [2]. Currently, the drugs for clinical treatment of NSCLC can be divided into three categories based on their mechanism of action: cytotoxic, molecularly targeted and immunotherapeutic drugs [3]. Cytotoxic drugs generally produce their antitumor efficacy by interfering with the synthesis of nucleic acids and/or proteins [4]. The drugs commonly used in the clinic to treat NSCLC are cisplatin (CDDP), vinorelbine, and paclitaxel [5]. The most effective treatment for NSCLC is CDDP, which activates apoptosis-related pathways by inducing DNA damage [6]. However, as chemotherapeutic treatment progresses, the likelihood of drug resistance increases, thereby decreasing the therapeutic efficacy of CDDP [7]. It has been hypothesized that multiple mechanisms are involved in mediating resistance to CDDP [8, 9]. It has been reported that CDDP resistance in NSCLC can result from alterations in: 1) alteration of influx and efflux of drugs from the cancer cells, such as copper transporter CTR1 [10, 11], P-gp [12] and other transporters [13, 14]; 2) enhancing the capacity for DNA repair, for instance, the upregulation of nucleotide excision repair related protein ERCC1 [15]; 3) downregulation of the expression of apoptosis proteins, including Bcl-2 and Bak [7, 16, 17]; 4) the change of important molecular signal pathways [11, 18, 19], such as IGF and MAPK pathways. Some strategies against cisplatin resistance have achieved initial results. For instance, Sen et al found that CHK1 inhibitor LY2606368 improved the response of platinum-resistant models to CDDP [20]. In addition, Socinski MA et al reported targeting components in the tumor microenvironment, such as immune system treatment, is associated with better survival outcomes [21]. Currently, however, the exact mechanisms underlying cisplatin resistance in NSCLC remain to be determined, and the drugs that have been developed to treat drug resistant lung cancer, based on our existing knowledge of drug resistance mechanisms, have not had significant therapeutic efficacy [8, 11]. Therefore, there is an urgent need to elucidate the mechanism of CDDP resistance in NSCLC and to develop novel and more efficacious drugs for lung cancer patients.

One approach in developing drugs to overcome CDDP resistance is to determine the expression of specific genes in these tumors. For example, high temperature requirement factor serine peptidase 1 (HtrA1) was the first identified member of the serine protease family [22, 23]. Recently, it has been reported that the down-regulation of HtrA1 promotes the survival, as well as the invasion and migration of cancer cells [24, 25]. Functional studies indicate that HtrA1 regulates a number of signaling pathways and protein substrates that mediate anti-tumor efficacy [22, 26]. HtrA1 is primarily involved in the regulating the following biological functions: the transforming growth factor-β (TGF-β) signaling pathway [27, 28], programmed cell death and apoptosis [29, 30], the EGFR/AKT pathway [31] and the inhibition of epidermal-interstitial transformation [25]. Studies have shown that the expression level of HtrA1 is negatively correlated to drug resistance and HtrA1 may be a target for drug development [32]. In NSCLC, the downregulation of HtrA1 mRNA and protein levels increases the number of tumor stem cell phenotypes in CDDP-resistant cells [33]. However, the characteristics, molecular mechanisms, and regulation of HtrA1 in CDDP resistance remain to be elucidated.

In this study, we identified HtrA1 as a tumor suppressor gene that was involved in cancer cell proliferation and migration, and in CDDP resistance in NSCLC cells. Furthermore, the mechanisms that downregulate HtrA1 in CDDP-resistant cells were elucidated, and a rescue strategy based on these regulatory mechanisms was devised to overcome CDDP resistance in NSCLC cells.

## Results

### The identification of HtrA1 as a cisplatin resistance-related gene in NSCLC cells

**Fig. 1:**
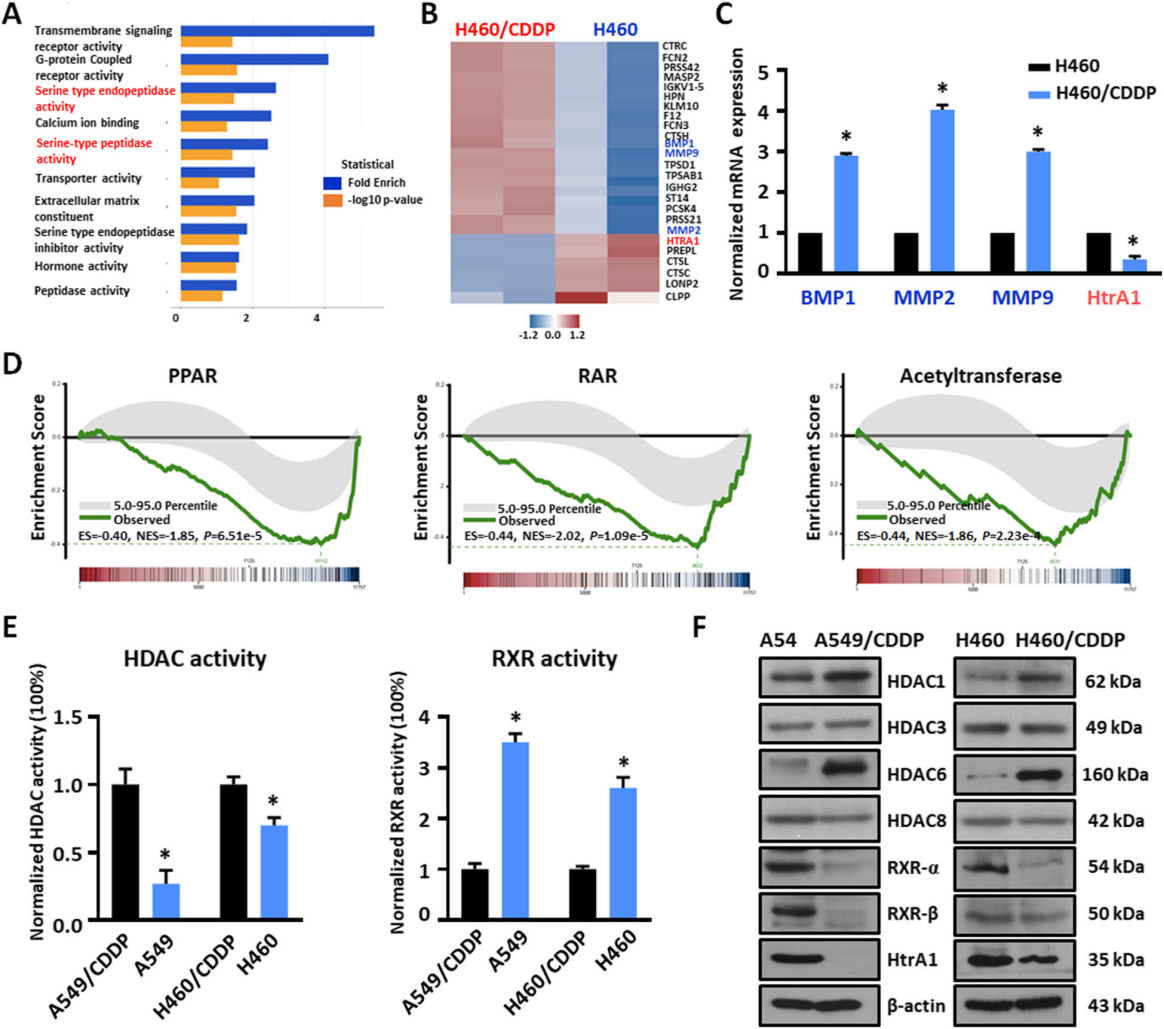
Identification of HtrA1 as a cisplatin resistance-related gene in NSCLC. **a**. The differential expression of genes in parental and NCI-H460/CDDP cells was compared using gene microarray analysis. The figure shows GO analysis (molecular function, MF) of the differentially expressed genes. Functions highlighted in red represent serine-type endopeptidase activity and serine-type peptidase activity. **b** and **c**. The differential expression of genes in NCI-H460 and NCI-H460/CDDP cells by **b**, gene microarray analysis and **c**, RT-PCR. **d**. GSEA analysis of PPAR, RAR and acetyltransferase activity in NCI-H460/CDDP cells. **e**. The activity of HDAC and RXR in CDDP resistant and parental cells. **f**. The protein expression levels of the main isoforms of HDAC and RXR in CDDP resistant cells and parental cells. ^*^P < 0.05, as compared to the parental cell group with the CDDP-resistant cell group

To determine the mechanisms that produce CDDP resistance in NSCLC cells, we performed gene microarray analysis in paired NSCLC and NSCLC-CDDP resistant cell lines. Spell out GO (GO) analysis data indicated that serine peptidase activity was significantly increased in NCI-H460/CDDP cells compared to the parental cells (Fig. 1a). Further analysis indicated that several oncogenes, such as MMP2, MMP9 and BMP1, were significantly upregulated in resistant cells (Fig. 1b). However, several genes were downregulated in CDDP resistant cells, including HtrA1, a cancer-related gene. The above data were confirmed by real-time RT-PCR analysis (Fig. 1c and Fig. S1A-B).

To elucidate the mechanism that regulates HtrA1 expression, we analyzed the promoter region of the HtrA1 gene using Transfac and JASPAR software. The results indicated that there were several binding sites for the nuclear receptor, RXR (data not shown). In addition, Gene Set Enrichment Analysis (GSEA) analysis indicated that the activity of PPAR and RAR, which form heterodimers with RXR [34], was significantly reduced in NCI-H460/CDDP cells and A549/CDDP cells (Fig. 1d and Fig. S1C). Furthermore, the acetyltransferase activity of NCI-H460/CDDP cells and A549/CDDP cells was also significantly decreased (Fig. 1d and Fig. S1C), suggesting that epigenetic regulation may be involved in this process. Next, we analyzed the activity of the epigenetic regulator, HDAC, and RXR, in three paired NSCLC cell lines. As shown in Fig. 1e and Fig. S1D, HDAC activity significantly increased, whereas RXR activity was significantly decreased in the resistant cell lines, consistent with the GSEA data. Furthermore, the expression of the HDACs proteins, including HDAC1 and HDAC6, was increased, whereas the expression of the RXR proteins was decreased, and the level of HtrA1 protein was significantly decreased in the CDDP resistant cell lines (Fig. 1f and Fig. S1E). To further clarify the role of HDACs in CDDP resistance, we treated NCI-H460/CDDP with HDAC6 specific inhibitors CG347B and pan-HDAC inhibitor SAHA. As shown in Fig. S1F, SAHA significantly increased the expression of HtrA1 in both mRNA and protein levels, while the HDAC6 inhibitor (CG347B) could not, suggesting that the total HDACs play a key role in HtrA1 regulation. Taken together, the above results indicate that HtrA1 is downregulated in CDDP resistant NSCLC cells, and the nuclear receptor RXR and the epigenetic regulatory enzyme HDAC may be involved in the regulation of HtrA1.

### The downregulation of HtrA1 by HDAC and RXR desensitizes NSCLC cells to CDDP

**Fig. 2:**
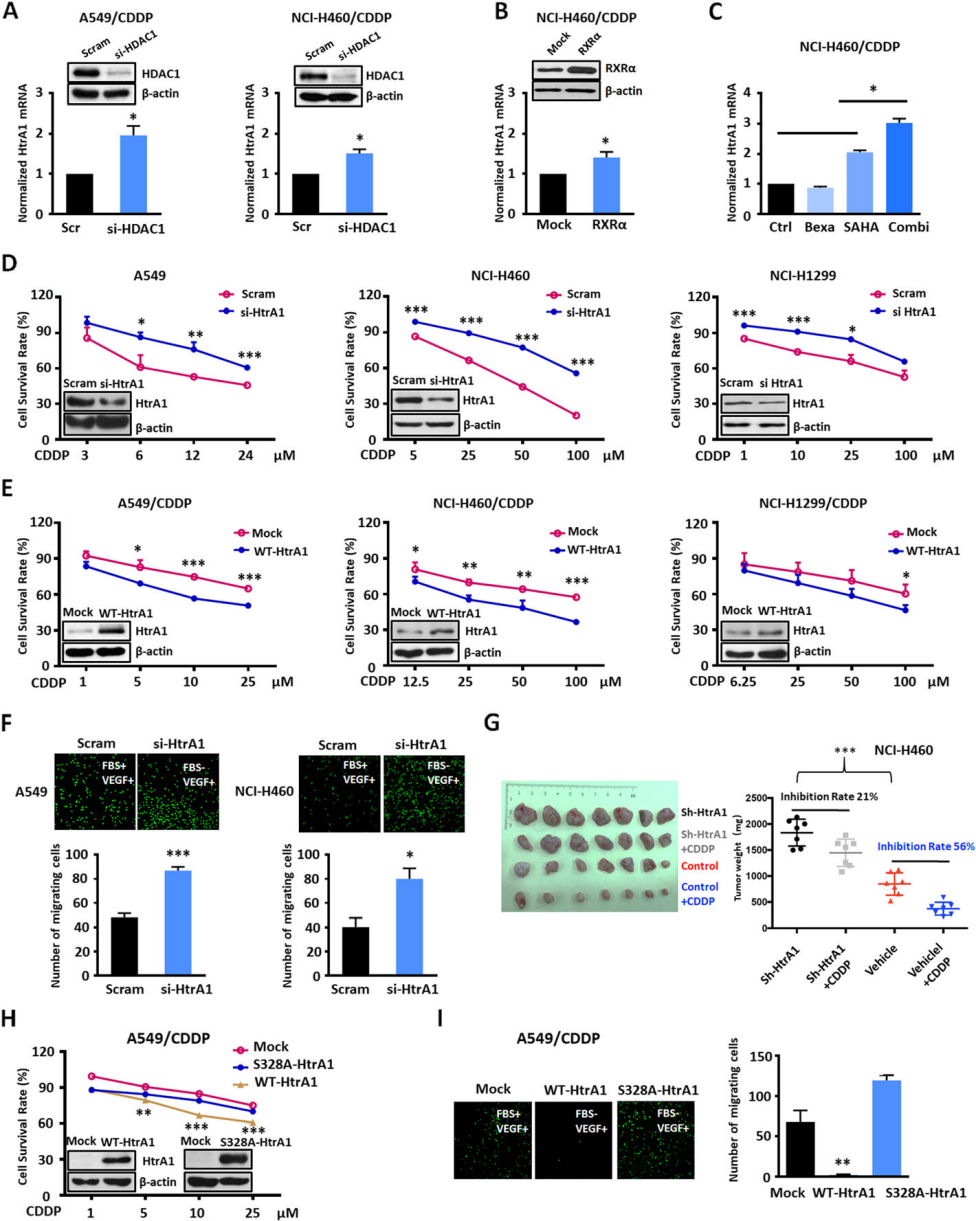
The downregulation of HtrA1 by HDAC and RXR increases the efficacy of cisplatin in NSCLC cells. **a**. RT-PCR analysis of HtrA1 in CDDP resistant NSCLC cells transfected with HDAC1 siRNA or control siRNA (Scramble). **b**. RT-PCR analysis of HtrA1 mRNA levels in the NCI-H460/CDDP cell line transfected with a RXRα overexpression or control plasmid. **c**. RT-PCR analysis of HtrA1 mRNA levels in NCI-H460/CDDP cells incubated with bexarotene (Bexa), SAHA or a combination of bexarotene and SAHA for 24 h. The final concentrations were: SAHA (5 μM), bexa (20 μM), SAHA+bexa (5 μM/20 μM). **d**. MTT assay results indicating the efficacy of CDDP in parental NSCLC cells transfected with HtrA1 siRNA or control siRNA for 48 h. **e**. MTT assay results indicating the efficacy of CDDP in CDDP resistant NSCLC cells transfected with HtrA1 overexpression or control plasmid for 48 h. **f**. Cell migration assay in parental NSCLC cells transfected with HtrA1 siRNA or control siRNA. **g**. The inhibitory efficacy of CDDP treatment on tumor weight in mice with NCI-H460 shHtrA1 and vehicle xenografts. **h**. MTT assay results indicating the CDDP sensitivity of A549/CDDP cells transfected with HtrA1 overexpression (S328A or WT) or control plasmid. **i**. Cell migration in A549/CDDP cells transfected with HtrA1 overexpression (S328A or WT) or control plasmid. ^*^P < 0.05, ^**^P < 0.01, ^***^P < 0.001, as compared to the scram or mock group

Next, we investigated whether RXR and HDAC affect HtrA1 expression and mediates CDDP resistance in NSCLC cells. We determined the HtrA1 mRNA levels in CDDP resistant NSCLC cells by transient silencing of HDAC1, the main isoform of HDAC. In so doing, HtrA1 mRNA levels were significantly increased in the HDAC1 siRNA group compared to the scramble control group (Fig. 2a and Fig. S2A). We also determined HtrA1 mRNA levels in drug resistant cells that overexpressed RXRα. The results indicated that HtrA1 mRNA levels were significantly increased in the RXRα-overexpressing cells compared to the control group (Fig. 2b and Fig. S2B). To determine the possible synergistic regulation of HtrA1 by HDAC and RXR, we measured HtrA1 expression in A549/CDDP and NCI-H460/CDDP cells incubated with the HDAC inhibitors SAHA and panobinostat (LBH-589) or the RXR agonist, bexarotene (Bexa), either individually or in various combinations. The combination of the HDAC inhibitors and Bexa significantly up-regulated HtrA1 expression compared to the control and single-compound groups in CDDP resistant NSCLC cells (Fig. 2c, and Fig. S2C), as well as parental cells (Fig. S2D-E). The co-regulation model was also demonstrated by gene manipulation. Combination of HDAC knockdown and RXR overexpression synergistically increase HtrA1 expression in CDDP resistant NSCLC cells (Fig. S2F-G). These results confirmed the co-regulatory effect of HDAC and RXR on HtrA1 expression in CDDP resistant NSCLC cells. To exclude whether there is a inter-regulation between HDAC and RXR, we detected the expression of them after treated with Bexa or SAHA. As shown in Fig. S2H, the expression levels of three RXR isoforms and HDAC1 did not change, suggesting their independent regulatory role. Next, we determined the efficacy of CDDP in paired NSCLC cells to CDDP after HtrA1 manipulation. Indeed, the efficacy of CDDP was significantly decreased in the parental NSCLC cells after the knock-down of HtrA1, whereas the efficacy of CDDP was significantly increased in CDDP-resistant NSCLC cells overexpressing HtrA1 (Fig. 2d-e).

To clarify the role of HtrA1, we performed migration assays in parental cells after transiently silencing HtrA1. Following the silencing of HtrA1, the migration of parental cells was significantly increased (Fig. 2f and Fig. S2I). Furthermore, the role of HtrA1 in anti-tumorigenesis and CDDP resistance was assessed by an in vivo study. Compared with the vehicle control group, the knockdown of HtrA1 in NCI-H460 cells resulted in a significant increase in tumor growth (Fig. 2g). In addition, in vivo data indicated that NCI-H460 xenograft tumors where HtrA1 was knocked down were more resistant to CDDP (inhibition rate 21%) than control NCI-H460 xenograft tumors without the knockdown of HtrA1 (inhibition rate 56%) (Fig. 2g).

To further determine if HtrA1’s efficacy in cisplatin resistance is dependent on its enzyme activity, we created a catalytically dead mutant of HtrA1 (S328A), and over-expressed the HtrA1 wild-type and dead-mutant in A549/CDDP cells. The efficacy of CDDP was significantly increased in the group expressing wild-type HtrA1, but not in the group expressing the dead-mutant (Fig. 2h). Similarly, in the A549/CDDP cell line, wild-type HtrA1 overexpression significantly reduced cell migration, whereas cell migration was not decreased in the dead-mutant (Fig. 2i).

### The relationship between HDAC, RXR and HtrA1 in NSCLC cases and their clinical significance in NSCLC

**Fig. 3:**
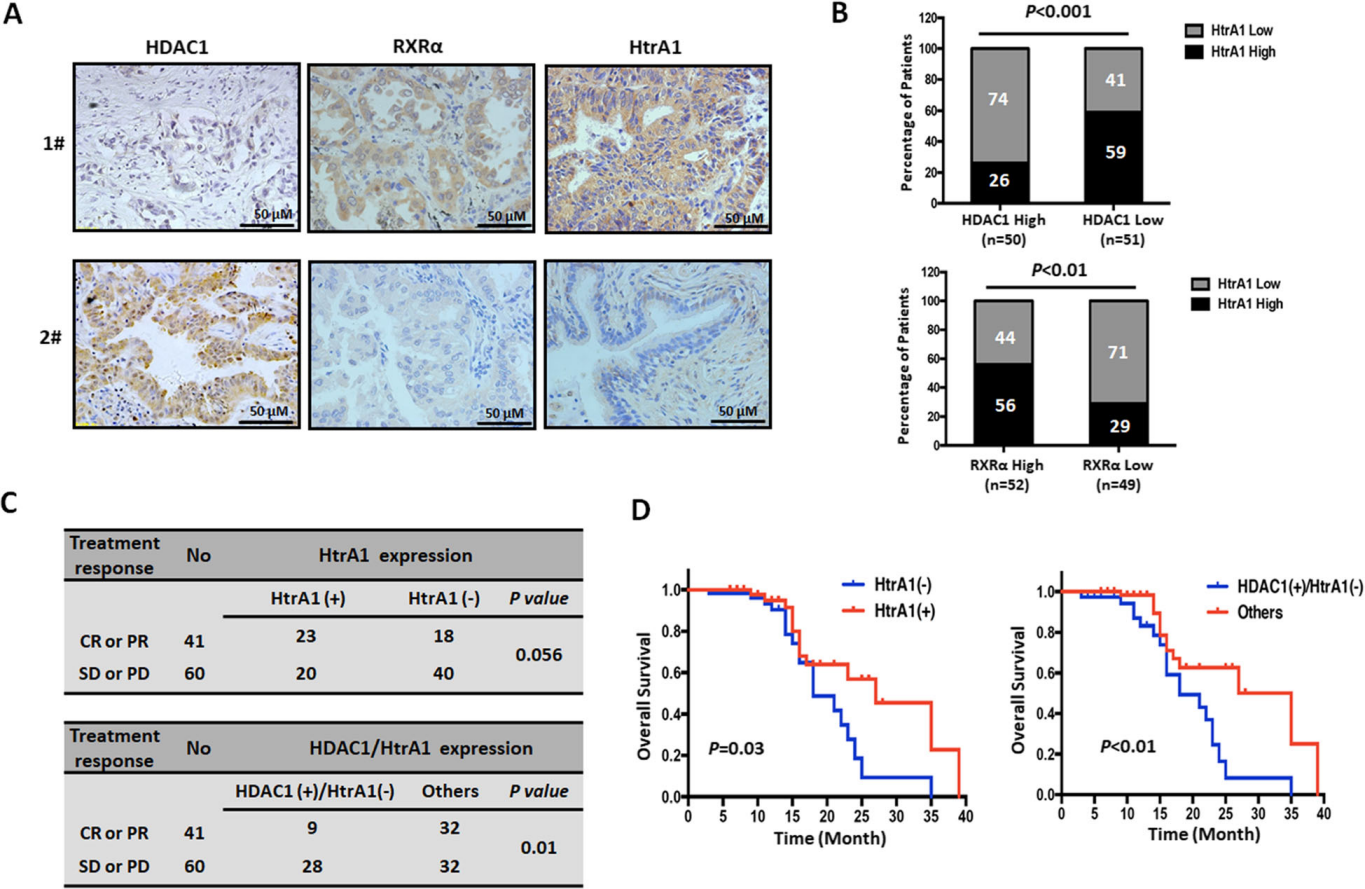
The relationship between HDAC, RXR and HtrA1 in NSCLC cases and their clinical significance in NSCLC. **a**. Representative sections of NSCLC tumor tissues from two patients treated with platinum. The expression of HDAC1, RXRα and HtrA1 was detected using immunohistochemistry. Patient 1# was HDAC1 low/RXRα high/HtrA1 high. Patient 2# was HDAC1 high/RXRα low/HtrA1 low. **b**. Statistical analysis of the expression patterns of HDAC1, RXRα and HtrA1 in tissue specimens from 101 NSCLC patients treated with platinum. **c** and **d**. Correlation of the expression of HtrA1 protein with **c** the treatment response and **d** the overall survival. CR, complete response; PR, partial response; SD, stable disease; PD, progressive disease

To confirm the relationship between HDAC, RXR, and HtrA1, we determined their expression levels in tissue specimens from 101 platinum-treated NSCLC patients using immunohistochemistry. The results indicated that 74% of patients with low levels of HtrA1 expression (*n* = 37) were in the high HDAC1 expression group (*n* = 50), whereas 59% of patients with higher HtrA1 expression (*n* = 30) were in the low HDAC1 expression group (*n* = 51). These results indicated that HDAC1 expression was negatively correlated with HtrA1 expression in platinum-treated NSCLC cases (*P* < 0.001, Fig. 3a and b). In contrast to HDAC1, RXRα expression was positively correlated with the expression of HtrA1 in NSCLC cases (*P* < 0.01, Fig. 3a and b). Furthermore, the downregulation of HtrA1 was non-significantly correlated with a poor response to treatment (*P* = 0.056, Fig. 3c) and significantly correlated with a poor overall survival (*P* < 0.05, Fig. 3d). Importantly, the opposite expression pattern (higher HDAC1 and lower HtrA1 expression levels) was significantly correlated with a poor treatment response and poor overall survival compared to the other NSCLC groups (Fig. 3c and d). Notably, the PROGgeneV2 tool (using the GSE30219 database, Fig. S3) also showed that the high HDAC1/low HtrA1 expression pattern was significantly correlated with a poor prognosis. Overall, the immunohistochemistry data further confirmed the interaction between HDAC1, RXRα, and HtrA1, and suggested that HtrA1, especially in combination with HDAC1, may be used as a predictive biomarker for platinum treatment response and prognosis in patients with NSCLC.

### The transcriptional activation of HtrA1 is dependent on heterodimeric RXRα complexes and HDAC activity

**Fig. 4:**
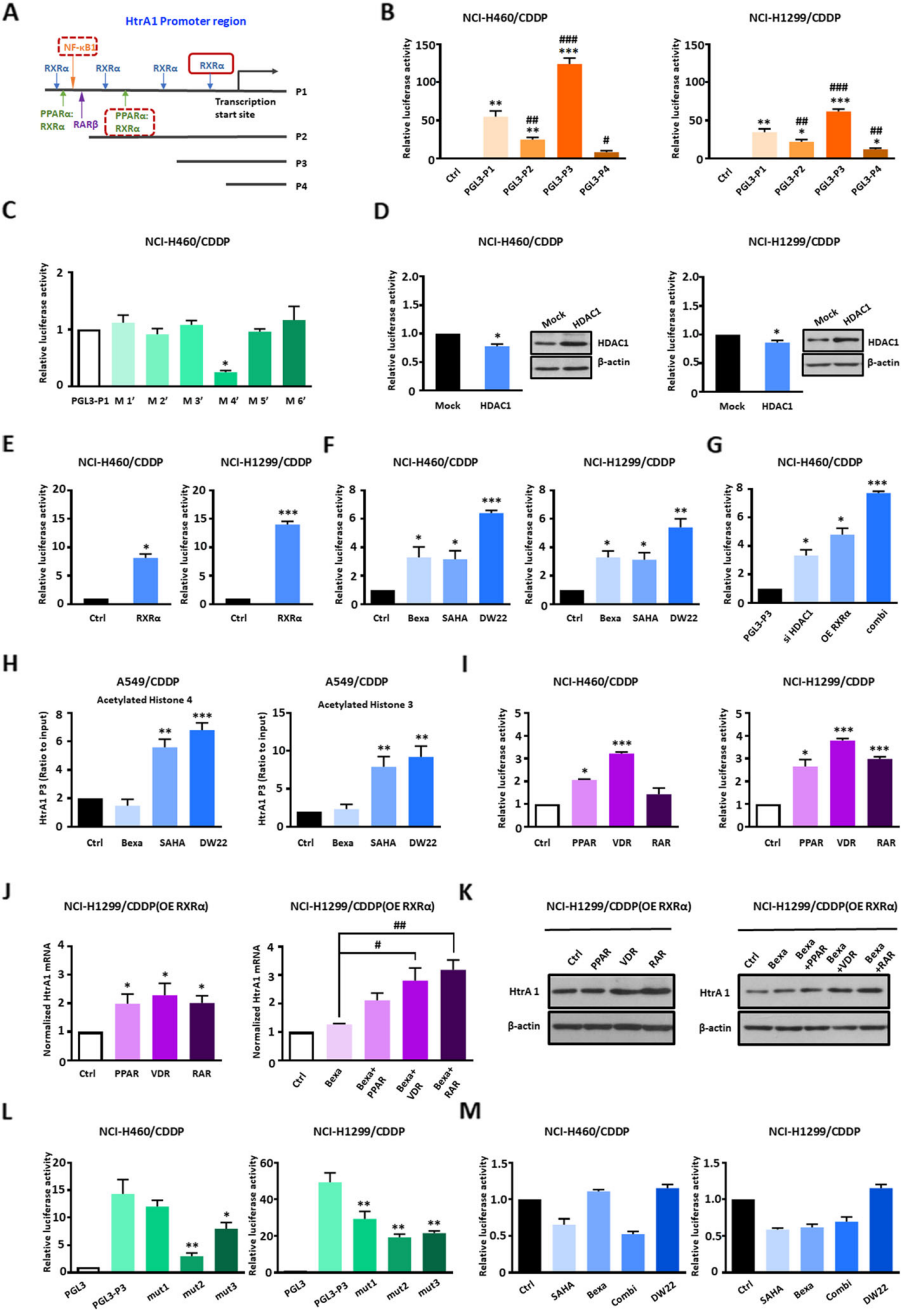
The transcriptional activation of HtrA1 depends on formation of RXRα heterodimers and HDAC activity. **a**. Schematic diagram of the HtrA1 promotor region (P1) showing the location of predicted binding sites for four different transcription factors, RXRα, RARβ, NF-κB1 and PPARα:RXRα. The promoter deletions (P2-P4) are shown below. **b**. Dual luciferase reporter assay for the activity of the four promoter constructs, pGL3-HtrA1-P1, P2, P3 and P4, in CDDP resistant NSCLC cells. ^#^*P* < 0.05, ^##^*P* < 0.01, ^###^*P* < 0.001, as compared to the PGL3-P1 group. **c**. Luciferase activity of the HtrA1 when mutated four RXRα binding sites in PGL3-HtrA1-P1. **d** and **e**. Luciferase activity elicited by the HtrA1 P3 promotor in CDDP resistant NSCLC cells after overexpression of HDAC1 and RXRα. **f** The luciferase activity of HtrA1 in CDDP resistant NSCLC cells when incubated with DW22 (20 μM), bexarotene (20 μM) and SAHA (5 μM) for 24 h. **g** Luciferase activity elicited by the HtrA1 P3 promotor in CDDP resistant NSCLC cells when silenced HDAC1, overexpressed RXRα and silenced HDAC1 and overexpressed RXRα simultaneously for 24 h. **h** The ChIP assay for the histone modifications involved in HtrA1. **i** Luciferase activity elicited by the HtrA1 P3 promotor in CDDP resistant NSCLC cells incubated with different heterodimer activators. **j** The expression of mRNA driven by the HtrA1 P3 promotor in RXRα-overexpressing NCI-H1299/CDDP cells when incubated with different heterodimer activators (left panel) and combinations of bexarotene with heterodimer activators (right panel). ^#^*P* < 0.05, ^##^*P* < 0.01, as compared to the bexarotene group. **k** The protein expression of HtrA1 in RXRα-overexpressing NCI-H1299/CDDP cells when incubated with different heterodimer activators (left panel) and combinations of bexarotene with heterodimer activators (right panel). **l** Luciferase activity elicited by the HtrA1 P3 promotor with mutations in the 3 RXRα binding sequences, pGL-HtrA1-P3-mut1, mut 2 and mut3 constructs. **m** Luciferase activity elicited by the HtrA1 P3-mut2 promotor, pGl3-HtrA1-P3-mut2 construct, in cisplatin-resistant NSCLC cells after incubation with SAHA, bexarotene, DW22 or SAHA+Bexarotene. ^*^P < 0.05, ^**^P < 0.01, ^***^P < 0.001, as compared to the control group or mock group or PGL3-P3 group

To further investigate the molecular mechanism of HtrA1 regulation, we used luciferase reporter assays to analyze transcription factor binding sites in the promoter of the HtrA1 gene. First, we constructed a series of reporter plasmids containing the full-length HtrA1 gene promoter (pGL3-HtrA1-P1) or with three deletions (pGL3-HtrA1-P2, P3, and P4; Fig.4a). We next determined the reporter gene activity in CDDP resistant and parental NSCLC cells using a dual luciferase reporter assay. Similar patterns of transcriptional activity were seen in both resistant cells and parental cells (Fig. 4b and Fig. S4A). The P3 region, which includes an RXRα binding site, had the highest activity in all four cell lines tested, suggesting that RXRα may act as a transcriptional activator in the HtrA1 promoter regulation. In addition, our data indicated that CDDP resistant cells had a higher reporter activity than the parental cells, providing further evidence that HtrA1 plays a role in CDDP drug resistance (Fig. S4B). To identify the functional binding site of RXRα, we mutated four RXRα binding sites alone or in different combinations in PGL3-HtrA1-P1. As shown in Fig. 4c and Fig. S4C, the luciferase activity of HtrA1 was significantly decreased when mutated M4’ site while no significant changes when mutated other sites or in combination, indicating RXRα binding site on P3 plays an important role in transcriptional activation of HtrA1. Next, to determine the epigenetic regulation of HtrA1, we overexpressed HDAC1 in NSCLC cells transfected with the pGL3-HtrA1-P3 reporter construct. The overexpression of HDAC1 in drug resistant cells weakly decreased luciferase activity, suggesting that HDAC1 regulate the transcriptional activity of the HtrA1 promoter to a certain extent (Fig. 4d). Similar results were obtained in parental cells (Fig. S4D). The overexpression of RXRα in both resistant and parental cells significantly increased the activity of the HtrA1 reporter construct, suggesting that RXRα activates HtrA1 transcription (Fig. 4e and Fig. S4E). To further investigate the transcriptional regulation of HtrA1 by RXR and HDAC, we measured pGL3-HtrA1-P3 reporter activity in resistant and parental cells incubated with bexarotene, SAHA, and DW22 (Fig. 4f and Fig. S4G). DW22 is a novel dual-target compound, previously discovered by our research group, that inhibits HDAC while activating RXR [35] (Fig. S4F). The results showed that the activity of the reporter was increased after incubation with SAHA or bexarotene in CDDP resistant cells (Fig. 4f). As expected, the activity after incubation with DW22 was significantly increased in higher degree (Fig. 4f and Fig. S4G). Besides, the fact that combination of HDAC knockdown and RXR overexpression synergistically increased the transcriptional activity of the HtrA1 promoter in CDDP resistant NSCLC cells confirmed the co-regulatory effect of HDAC and RXR on HtrA1 (Fig. 4g). The ChIP assay was performed to assess epigenetic regulation, including Acetylated Histone 3 and Histone 4, and the ability of the transcription factor, RXRα, to bind to specific sites on the HtrA1 promoter fragment P3. The results indicated that similar with SAHA, DW22 also increased directly binding of the acetylated H4 and acetylated H3 to HtrA1 promoter in drug resistant cells (Fig. 4h). Also, the ability of RXRα’s binding to HtrA1 promoter was enhanced by DW22 in drug resistant cells (Fig. S4H). The above data confirmed the synergistic regulation of HtrA1 by HDAC and RXR.

RXR forms homodimers with itself or heterodimers with the retinoic acid receptor (RAR), vitamin D receptor (VDR) and peroxisome proliferator-activated receptor (PPAR), to regulate signal transduction pathways involved in processes such as cell proliferation, differentiation, metabolism and apoptosis [9]. Therefore, we incubated resistant and parental cells with pioglitazone, a PPAR agonist, calcitriol, a VDR agonist and tamibarotene, a RAR agonist, to verify whether the regulatory function of RXR is dependent on a heterodimeric transcriptional complex. All three of the aforementioned agonists significantly increased HtrA1 reporter gene activity in NCI-H1299/CDDP cells, with calcitriol producing the greatest increase, at the same time, VDR agonist calcitriol also significantly increased HtrA1 reporter gene activity in other cells. (Fig. 4i and Fig. S4I). Thus, RXR may depend on the formation of heterodimeric transcriptional complexes to regulate the HtrA1 gene. Interestingly, there was no significant change in HtrA1 mRNA and protein expression in both parental cells and resistant cells following treatment with the heterologous agonists, pioglitazone, calcitriol and tamibarotene (Fig. S4J and Fig. S4K). We also combined RXR homologous agonists with heterologous agonists and determined their effect on the regulation of HtrA1 mRNA expression. However, the activity of the reporter construct was increased slightly, indicating that only the combination of agonists did not dramatically increase the level of HtrA1 (Fig. S4L). Consequently, we hypothesized that endogenous RXRα does not affect the transcription process of HtrA1 gene in the presence of an additional agonist.

Next, we determined the levels of HtrA1 mRNA and protein in RXRα-overexpressing NCI-H1299/CDDP cells incubated with heterodimeric agonists and bexarotene (Fig. 4J and Fig. 4K). We found that bexarotene/RAR was potent and bexarotene/VDR was medium, while bexarotene/PPAR showed a weak effect on the expression of HtrA1. These data suggest that RXRα plays an important role in the transcriptional activation of HtrA1, and this is dependent on the formation of a heterodimer. In order to confirm the role of RXRα in the regulation of HtrA1, we mutated the RXRα binding sites within the HtrA1 promoter fragment P3 to create the reporter constructs, pGL3-HtrA1-P3-mut1, mut2 and mut3 (Fig. S4M). We then determined the transcriptional activity of these reporters in cells incubated with a RXRα agonist, Bexarotene and a HDAC inhibitor, SAHA. The luciferase activity of the pGL3-HtrA1-P3-mut2 was decreased in all cells, suggesting that HtrA1 transcription activity is dependent on RXRα (Fig. 4l and Fig. S4N). Importantly, there were no significant effects on HtrA1 transcriptional activity in resistant cells and parental cells transfected with pGL3-HtrA1-P3-mut2 plasmid after incubation with SAHA, bexarotene and DW22 (Fig. 4m and Fig. S4O). The regulation of HtrA1 by RXR and HDAC was further confirmed by ChIP assay to detect RXRα binding. As shown inFig. S4P, we found overexpression of RXRα led to increase RXRα binding to HtrA1 promoter, and treatment with Bexa, SAHA and DW22 could further promote RXRα binding. On the contrary, mutation in mut2 point could result in the binding reduction. Similary, co-expression HDAC1 also contribute to decrease of RXRα binding. The Overall, the results suggest that RXR is an important transcriptional activator of the HtrA1 gene, whereas HDAC contributes to transcription repression of HtrA1 gene.

### The dual-target compound, DW22, mediates its anti-cancer efficacy in cisplatin-resistant cells by regulating HtrA1 expression

**Fig. 5:**
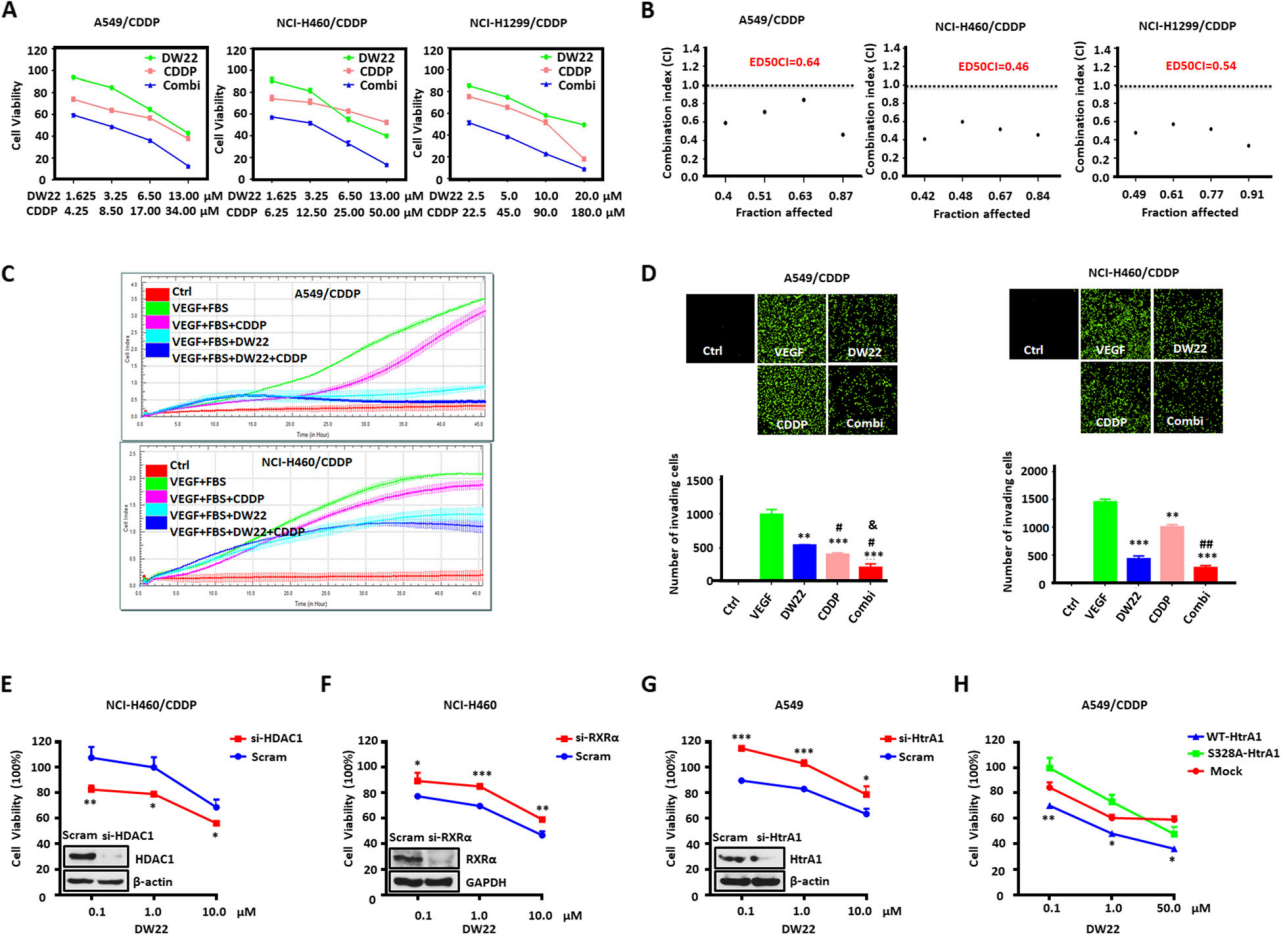
The dual-target compound DW22 significantly inhibits the growth of cisplatin-resistant cells by regulating HtrA1 mRNA levels. **a** MTT assay indicating the efficacy of cisplatin in cisplatin-resistant NSCLC cells following incubation with DW22, cisplatin and DW22 + cisplatin for 72 h. DMSO was set as the control group that comparing to treatment groups. **b** The combination index for DW22 + cisplatin in cisplatin-resistant NSCLC cells was calculated using the Calcusyn program. CI < 0.90 indicates synergism, 0.90–1.10 indicates an additive effect and > 1.10 indicates antagonism. **c**, **d** The inhibitory effects of DW22 and CDDP, separately and together, on **C** migration and **d** invasion in cisplatin-resistant NSCLC cells. The final concentrations were: CDDP (5 μM), DW22 (13 μM), CDDP+DW22 (5 μM/13 μM) **e-h** MTT assay data indicating the sensitivity to DW22 in **e** H460/CDDP cells transfected with HDAC1 siRNA or control siRNA, **f** H460 cells transfected with RXRα siRNA or control siRNA, **g** A549 cells transfected with HtrA1 siRNA or control siRNA, and **h** A549/CDDP cells transfected with a HtrA1 overexpression (S328A or WT) or control plasmid. ^*^P < 0.05, ^**^P < 0.01, ^***^P < 0.001, as compared to the VEGF group or scramble or Mock group. ^#^P < 0.05, ^##^P < 0.01, as compared to the DW22 group. ^&^P < 0.05, as compared to the CDDP group

Our experiments provide information about the molecular mechanisms by which HDAC and RXRα regulate the expression of HtrA1, and how the HDAC/RXR/HtrA1 signaling axis affects the efficacy of CDDP in NSCLC cells. Therefore, we hypothesized that suppressing this signaling axis may represent a potential approach for the reversal of CDDP resistance. As shown in Fig. 5a and b, the combination of DW22 and CDDP had a synergistic effect on the inhibition of cell growth. Compared to the parental cells, the synergistic effect was greater in NCI-H460/CDDP and NCI-H1299/CDDP cells (Fig. S5A-B). Typically, resistant cells have an increased capacity for invasion and migration compared to parental cells, and this was confirmed by our results (Fig. S5C). Single treatment in CDDP resistant cells with SAHA, LBH-589 or Bexarotene moderately inhibited the invasion and migration ability, and the inhibitory effect was more significant when SAHA, LBH and Bexarotene were used simultaneously or when DW22 was used (Fig. S5D-G). These results indicate that dual targeting of HDAC and RXR increases the inhibition of cell migration and invasion. Notably, the combination of DW22 and CDDP produced a significant inhibition of migration (Fig. 5c) and invasion (Fig. 5d) in CDDP-resistant NSCLC cells compared to incubation with only one compound.

Next, we conducted additional experiments to determine the role of the HDAC/RXR/HtrA1 signaling axis in mediating the efficacy of DW22 to reverse resistance to CDDP. As shown in Fig. S5H-I, the mRNA and protein expression levels of HtrA1 were enhanced by DW22 treatment in NCI-H460/CDDP resistant cells. Next, we determined the efficacy of DW22 when HDAC, RXR and HtrA1 were silenced, or when HtrA1 was overexpressed. NCI-H460/CDDP cells became more sensitive to DW22 to reverse resistance to CDDP after transfection with HDAC1 siRNA, whereas the opposite effect occurred in NCI-H460 cells after transfection with RXRα siRNA (Fig. 5e-f). To determined the effect on HtrA1, we knocked down HtrA1 in A549 cells, and then incubated the cells with DW22. The result indicated that DW22 was less efficacious in the HtrA1 knockdown group compared to the scramble control (Fig. 5g). Next, we overexpressed HtrA1 in A549/CDDP cells. As predicted, the overexpression of wild-type HtrA1 in A549/CDDP cells increased the sensitivity to DW22 (Fig. 5h). Interestingly, overexpressing the dead-mutant (S328A) HtrA1 in A549/CDDP had no significant effect on the cell sensitivity to DW22 compared to mock cells. Thus, HtrA1-induced sensitivity to DW22 requires the enzymatic activity of HtrA1. These results show that the dual targeting of HDAC and RXR by DW22 can reverse CDDP resistance in NSCLC, and this reversal was dependent on the HDAC/RXR/HtrA1 signaling axis.

### DW22 decreases CDDP resistance in NSCLC by rescuing HtrA1 in vivo

**Fig. 6:**
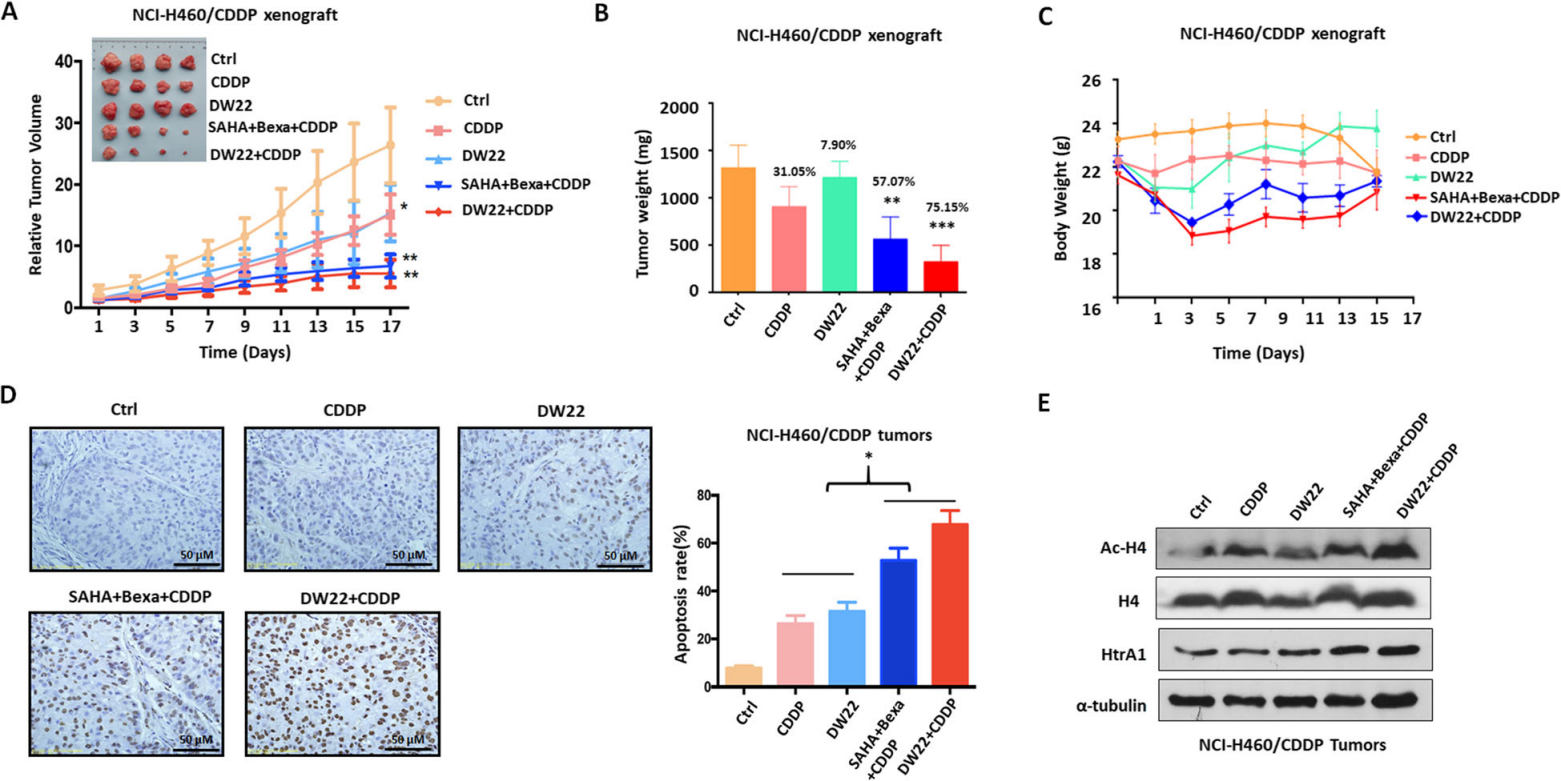
DW22 significantly decreases cisplatin resistance in NSCLC cells in vivo by rescuing HtrA1. **a-c** The effect of cisplatin, DW22 and cisplatin + DW22 on **a** relative tumor volume, **b** tumor weight and **c** body weight in Balb/c-nu mice xenografted with NCI-H460/CDDP cells. **d** TUNEL staining of NCI-H460/CDDP xenografted tumors. **e** Western blot analysis of H4, Ac-H4 and HtrA1 expression in NCI-H460/CDDP xenograft tumors from Balb/c-nu mice. ^*^P < 0.05, ^**^P < 0.01, ^***^P < 0.001, as compared to the the control group

We conducted experiments to validate the above in vitro results using an in vivo model. We used NCI-H460/CDDP and A549/CDDP xenograft models to determine the inhibitory efficacy resulting from targeting HDAC/RXR/HtrA1 in vivo. NCI-H460/CDDP or A549/CDDP cells were injected into the flanks of male nude mice. In the NCI-H460/CDDP mice treated with CDDP or DW22 alone, the tumors were significantly larger and greater in weight compared to tumors in mice treated with DW22 and CDDP (Fig. 6a-b). Interestingly, treatment with SAHA, bexarotene and CDDP also significantly reduced NCI-H460/CDDP tumor growth, further confirming that the dual targeting of HDAC and RXR reverses CDDP resistance. Furthermore, the combination of DW22 and CDDP also significantly reduced the tumor burden in the A549/CDDP xenograft model (Fig. S6A). The representative images of tumor were exhibited in Fig. 6a and Fig. S6A. Moreover, there was no significant change between CDDP group and combined treatment group in the body weight and viscera index in the both xenograft mice model (Fig. 6c, Fig. S6B, C). TUNEL staining (Fig. 6d) also confirmed that SAHA/bexarotene/CDDP and DW22/CDDP significantly induced cell apoptosis (TUNEL-positive cells). In addition, the pro-apoptosis action in the combination of DW22 and CDDP treatment was further confirmed by the increase of Bax, cleaved PARP, cleaved Caspase3 and decrease of Bcl-2 (Fig. S6D) in A549/CDDP tumor tissues. Western blot data also indicated that the increased the expression of HtrA1 by up-regulating the level of acetylated Histone4 in SAHA/bexarotene/CDDP and DW22/CDDP treated NCI-H460/CDDP tumor tissues (Fig. 6e, Fig. S6E). It is possible that these results are due to the dissociation of DNA that is facilitated by the acetylation of Histone4, producing an opening of the chromatin, thereby increasing the transcription of tumor suppressor genes. The above data indicate that DW22 can reverse CDDP resistance in two xenograft models by inducing cell apoptosis and upregulating HtrA1 expression.

## Discussion

NSCLC, the most common type of lung cancer, produces significant morbidity and mortality [2]. CDDP is one of the most effective treatments for NSCLC, but CDDP-treated patients are usually prone to developing drug resistance [8, 9]. The mechanisms of CDDP resistance have been explored for decades, and recent research indicates that a decrease in HtrA1 expresison is correlated with drug resistance [32, 36]. In this study, our gene microarrays and bioinformatics data indicated that the expression of HtrA1 and RXR were downregulated and HDAC expressed was upregulated in CDDP-resistant NSCLC cells. HtrA1 mRNA levels were significantly increased when HDAC1 was transiently silenced or RXRα was overexpressed in CDDP-resistant NSCLC cells. Based on previous results and our present data, we hypothesized that the downregulation of HtrA1 was significantly correlated with HDAC overexpression and RXR downregulation in CDDP-resistant NSCLC cells. Our hypothesis was substantiated by data indicating the incubation of CDDP-resistant NSCLC cells with the HDAC inhibitor, vorinostat and the RXR agonist, bexarotene, significantly increased the levels of HtrA1. Given our results indicating that HtrA1 expression is involved in mediating CDDP resistance, we transiently silenced HtrA1 in the parental cells and overexpressed HtrA1 in CDDP-resistant cells. The CDDP sensitivity and invasion ability were increased in the parental cells,while were decreased in CDDP-resistant cells. Finally, our in vivo xenograft data indicated knockdown of HtrA1 in NCI-H460 cells resulted in a significant increase in tumor growth (see Fig. 2).

Recent studies indicate that epigenetic mechanisms associated with abnormal regulation of gene expression occur frequently in NSCLC tumors [37]. Epigenetic changes are involved in the development and progression of tumors and may contribute to the development of resistance by interfering with tumor growth regulation pathways and proapoptotic programs [38]. We previously reported that histone deacetylase (HDAC) was activated in paclitaxel-resistant NSCLC cells, increasing proliferation and tumorigenesis of paclitaxel-resistant NSCLC cells in vitro and in vivo [39]. In addition, we have shown that CDDP played a role in the increased activity of HDAC, and the combination of vorinostat and cisplatin produced a 1) synergistic anti-cancer efficacy in NSCLC cell lines in a TRIB1-dependent manner and 2) significant decrease in tumor size and weight in mouse xenograft models [40]. These findings indicated that HDAC expression is significantly positively correlated with drug resistance in tumor cells, and its expression level and activity are significantly increased in drug-resistant cells and negatively correlated with the prognosis of NSCLC patients [41]. Recently, it has been reported that HDAC inhibitors such as vorinostat and panobinostat, which are approved by the FDA, can reverse the malignant phenotype of CDDP-resistant NSCLC and when used with CDDP, they inhibit the growth of NSCLC [42, 43], which further demonstrate the potential value, as a anti-drug resistance strategy, based on HDAC therapy. Furthermore, it also should be noted that there are several HDAC subtypes involved into development and progression in cancer. In the present study, we found HDAC1 and HDAC6 were upregulated in CDDP-resistant NSCLC cells, but not HDAC3 and HDAC8, suggesting that the expression of specific HDACs are positively correlated with CDDP resistance.

Previously, it has been reported that target genes can be activated by activating the associated signaling pathway [44] or by directly activating the nuclear receptor [45]. NSCLC resistance studies have shown that RXR agonists are efficacious in reversing NSCLC resistance to gemcitabine and paclitaxel by blocking the amplification of specific drug resistance genes [46, 47], suggesting that RXR activity plays a role in mediating the resistance of NSCLC cells to certain drugs. Here, we first identified a novel signaling axis, consisting of the histone deacetylation enzyme (HDAC), the nuclear receptor, RXR, and the serine protease HtrA1, that were involved in the progression of CDDP resistance. In the parental and CDDP-resistant cells, the overexpression of HDAC1 had just slightly regulatory effect alone on HtrA1 transcription. In addition, given pan-HDAC inhibitor could obviously upregulate the expression of HtrA1, whereas specific inhibitor could not done it, suggesting regulation of HtrA1 is dependent on the co-regulation of HDAC subtypes. Notably, HtrA1 activity was significantly increased when RXRα was overexpressed, indicating that RXRα transcriptional activates HtrA1. Furthermore, the compound DW22, which is an HDAC inhibitor and RXR agonist [33], increased HtrA1 activity in a concentration - dependent manner. These results suggest that the upregulation of HtrA1 is dependent on HDAC and RXR regulation. A possible mechanistic explanation for this is that the inhibition of HDAC, which increased the likelihood of chromatin being in an open or relaxed conformation, increasing the accessibility of transcription factors to DNA, facilitated the binding of the transcription factor RXR to its specific target sites in the HtrA1 promoter. RXR can form homodimers or heterodimers with the protein, retinoic acid receptor (RAR), vitamin D receptor (VDR), thyroxin receptor (TR), peroxisome proliferator-activated receptor (PPAR) and neuro-inducing receptor (NGFIB) [34]. These homo/heterodimers participate in signal transduction pathways that regulate processes such as cell proliferation, differentiation, metabolism and apoptosis [48, 49]. We incubated CDDP-resistant cells with the RXR homologous agonist, bexarotene and the heterologous agonists, pioglitazone (PPAR agonist), calcitriol (VDR agonist) and tamibarotene (RAR agonist) to determine if RXR regulation was dependent upon homodimeric or heterodimeric transcription complexes. Our results indicated that HtrA1 activity was significantly increased when bexarotene was used in combination the VDR and RAR agonists in RXRα-overexpressing H1299/CDDP cells. These results indicated that RXR is an important target for the regulation of HtrA1 gene transcription, and the activity of RXR is dependent on the formation of RXR:VDR and RXR:RAR heterodimers.

The rescue of tumor suppressor genes is one of the strategies for the treatment of cancerous tumors [50] Several methods for rescuing tumor suppressor genes have been reported, such as generating an overexpressor [51] and targeting apoptosis and necroptosis pathways [52]. However, these genetic intervention strategies are often problematic, and there have only been a few reports on the coordinated regulation of epigenetic enzymes and nuclear receptors. Therefore, we determined if CDDP resistance could be reversed by rescuing HtrA1. Our results indicated that the inhibition of HDAC and simultaneous activation of RXR increases the expression of HtrA1, thus increasing the response of CDDP-resistant NSCLC cells to cisplatin and inhibiting the migration and invasion of CDDP-resistant cells. Furthermore, the inhibition of HDAC and simultaneous activation of RXR significantly enhanced the efficacy of cisplatin by increasing HtrA1 expression in CDDP-resistant NSCLC xenograft tumors in vivo.

## Conclusion

**Fig. 7:**
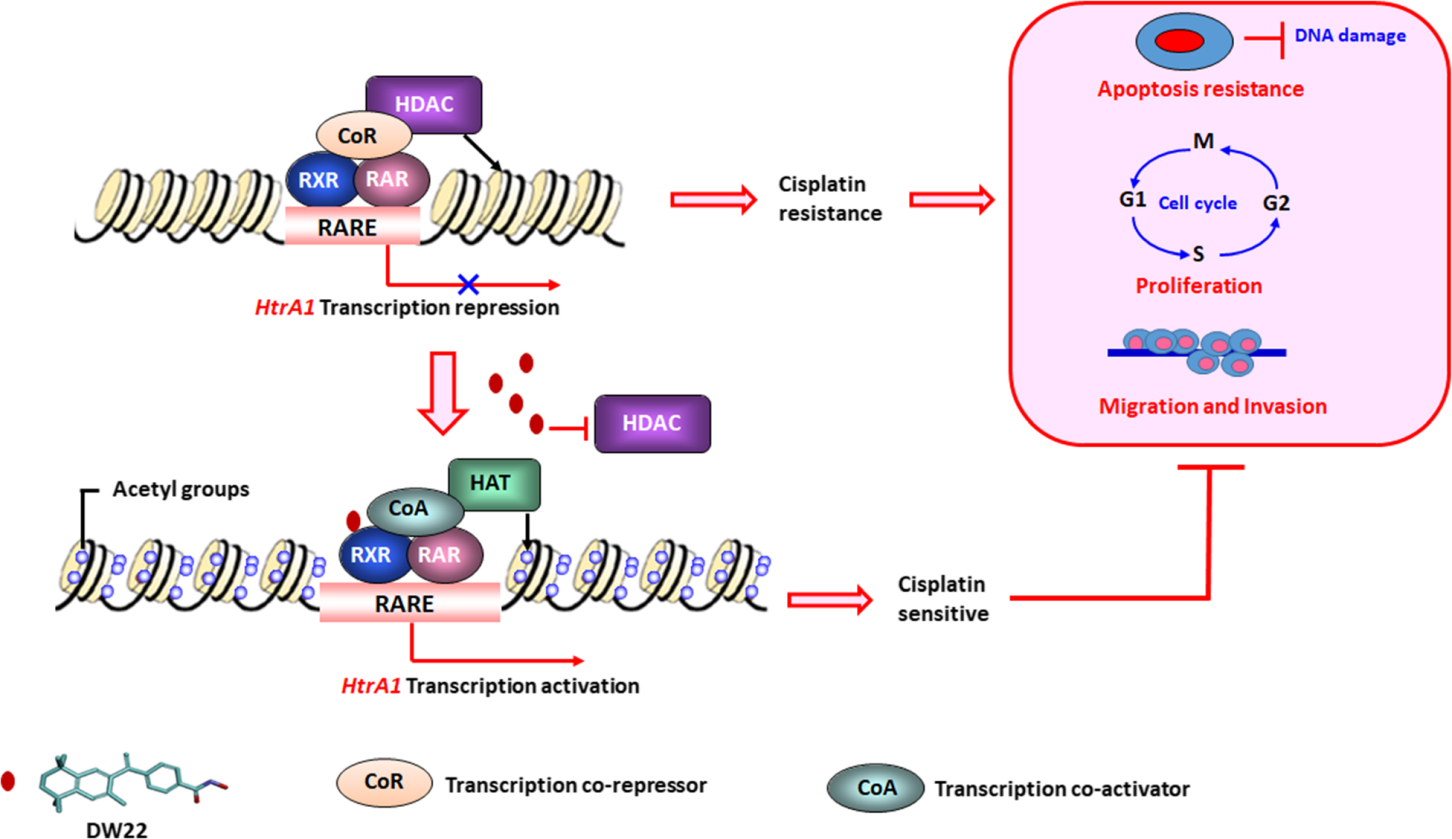
Schematic diagram. Regulation of HtrA1 by HDAC and RXR significantly increases the efficacy of cisplatin in NSCLC/CDDP resistant cells

The findings of our study are summarized in a schematic diagram (Fig. 7). In CDDP-resistant NSCLC, HDAC and RXR synergistically regulate the expression of HtrA1. The inhibition of HDAC and simultaneous activation of RXR up-regulate HtrA1 expression, and this signaling axis is involved in mediating in vitro and in vivo resistance to cisplatin. Mechanistically, RXRα is an important transcriptional activator of HtrA1, and it activates HtrA1 transcription by forming heterodimers. The efficacy of CDDP was increased in CDDP-resistant cells by DW22, which by inhibiting HDAC and activating RXR, significantly decreased the invasion and migration of tumor cells and inhibited the growth of xenograft tumors, reversing cisplatin resistance. The results of this study reveal a new strategy to rescue a tumor suppressor gene, which may provide a breakthrough for the discovery of novel drugs that are efficacious in overcoming chemotherapeutic resistance.

## Methods

### Cell culture

The human NSCLC cell lines A549, NCI-H460 and NCI-H1299 (American Type Culture Collection, Manassas, VA, USA) were grown in RPMI 1640 medium (Gibco, USA), with 10% fetal bovine serum (Gibco, USA) and 1% penicillin-streptomycin (Gibco, USA) at 37 °C in a 5% CO_2_ incubator. The same cell lines with acquired cisplatin resistance, in brief, NCI-H460 and NCI-H1299 cells were induced by CDDP from 500 ng/ml at the beginning and increased to 1500 ng/ml step by step until the RI value were enough to identify the resistance. The final IC_50_ and RI values were shown in supplementary Table 1. A549/CDDP (obtained from KeyGEN BioTECH, China), NCI-H460/CDDP and NCI-H1299/CDDP (constructed in our lab), were maintained by continuous exposure to a medium containing 1000 ng/ml of CDDP for A549/CDDP and 1500 ng/ml of CDDP for NCI-H1299/CDDP and NCI-H460/CDDP.

### Compounds

DW22 (MV: 340.68) was synthesized in our lab as previously described [35]. Cisplatin (CDDP), panobinostat (LBH-589), CG347B, and bexarotene were obtained from MedChem Express (Monmouth Junction, NJ, USA). Vorinostat (SAHA) was obtained from Sigma-Aldrich (St. Louis, MO, USA).

### Cell viability assay

In vitro cell viability was determined using the MTT assay. Cells (6 × 10^3^/well) were seeded in 96-well culture plates. The cells were incubated with various concentrations of the test compounds for 72 h at 37 °C in a 5% CO_2_ incubator, after which 10 μl of the MTT solution (5 mg/ml) was added to each well, and the plates were incubated for an additional 4 h at 37 °C. Subsequently, 100 μl of DMSO was added to each well and the optical density of each well was measured at 570 nm using a multi-mode plate reader (Molecular Devices, San Jose, CA, USA).

### Transwell invasion assays

Cell invasion was assessed using Transwell Permeable Supports (Corning, NY, USA). The chemoattractant VEGF in the lower chambers consisted of 500 μl of medium containing 10% fetal bovine serum with different concentrations of the test compounds. Approximately 4 × 10^5^–1 × 10^6^ cells/ml were resuspended in 100 μl serum-free medium and plated onto Transwell filter inserts coated with Matrigel 1:8 (BD Biosciences, Franklin Lakes, NJ, USA) for the invasion assay. Cells on the bottom side were fluorescently labelled with calcein-AM and photographed using a ImageXpress-Micro high content system (Molecular Devices, San Jose, CA, USA).

### Real-time cell analysis (RTCA)

The assays were performed with cell invasion migration plates that contained 16 modified Boyden chambers and detection of cell invasion using a xCELLigence Analyser System (ACEA Biosciences, San Diego, CA, USA). The experiments were conducted according to the manufacturer’s instructions, with the membrane uncoated for migration assays. A chemotactic signal for cell migration was provided by inoculating 30,000–50,000 cells in the serum-free medium in the upper chamber and supplying 10% FBS in the lower chamber fetal bovine serum.

### Western blotting

Cell lysates were extracted in RIPA buffer (CST, Danvers, MA, USA) and protein separation was performed using electrophoresis in 8–10% arc-bis gels. The proteins were transferred onto polyvinylidene difluoride membranes by a transfer system (Bio-Rad, Hercules, California, USA). The membranes were incubated with the appropriate primary and secondary antibodies, and then reacted with ECL detection reagents (Thermo Fisher Scientific, Waltham, MA, USA) and incubated for several minutes in a dark room. All antibody information is shown in supplementary Table 2.

### Immunohistochemistry and TUNEL assay

Clinical tissue samples were embedded in paraffin and antigen retrieval was performed. Following the blockade of endogenous peroxidase activity, the samples were incubated with the primary antibodies of interest and the appropriate secondary antibodies and reacted with DAB detection reagents. The immunoreactive staining of proteins in tumor tissue was scored by applying a semi-quantitatively immunoreactive scoring (IRS) system. Category A documented the intensity of immunostaining as 0 (no immunostaining), 1 (weak immunostaining), 2 (moderate immunostaining), and 3 (strong immunostaining). Category B documented the percentage of immunoreactive tumor cells as 0 (none), 1 (< 25%), 2 (26–50%), 3 (51–74%), and 4 (> 75%). Multiplication of category A and B resulted in an IRS ranging from 0 to 12 for each tumor. The median value of the immunoreactive score was chosen as the cut-off criterion to dichotomize into high- and low-expression subgroups.

The TUNEL system (Roche, Basel, Switzerland) was used to detect apoptosis in tumor sections on slides according to the manufacturer’s protocol. The TUNEL reaction solution was substituted with TdT-free solution to create a negative control. The sections were incubated for 10 min with DNase and visualized using DAB staining. Positive nuclei were identified basd on the presence of a brown color. The percentage of positive cells out of the total cells counted was calculated.

### RNA isolation and RT-PCR

Total RNA was extracted using a Trizol Reagent (Thermo Fisher Scientific, USA) and cDNA was synthesized using a Revert Aid First Strand c DNA Synthesis Kit (Thermo Fisher Scientific, USA), according to the manufacturer’s protocol. Quantitative RT-PCR analyses were performed in technical triplicates using a SYBR Green Supermix kit (Thermo Fisher Scientific, USA). The expression levels of HtrA1 were determined using the 2–ΔΔCt method and normalized to the housekeeping genes β-actin or GAPDH. All primers sequences are shown in supplementary Table 3.

### Vectors and transfections

The pcDNA plasmid was a gift from Professor Chio Oka (NARA Institute of Science and Technology, ikoma, Japan), the pCMX-hRXR-α and pBJ5-HDAC plasmids were a gift from Professor Makoto Makishima (Nihon University, Tokyo, Japan), and the pGL3-basic and phRL-tk plasmids were obtained from Yingrun Biotechnologies (Changsha, China). HDAC1, RXRα, HtrA1 and control siRNA were obtained from Life Technologies (Waltham, MA, USA), RXRγ siRNA was obtained from RIBOBIO (Guangzhou, China). Cells were transfected with plasmids using Lipofectamine 3000 (Thermo Fisher Scientific, Waltham, MA, USA) for 48 h and siRNA using Lipofectamine RNAi MAX (Thermo Fisher Scientific, Waltham, MA, USA) for 24 h, followed by the next treatment.

### Luciferase reporter assay

The activity of the HtrA1 promotor was determined using a dual-luciferase reporter assay system, according to the manufacturer’s instructions (Promega, Madison, WI, USA). The four promoter regions (P1-P4) of the HtrA1 gene were cloned into the pGL3-basic vector.

The RXRα transcription factor binding sites were mutated based on a consensus nucleotide sequence in the HtrA1 P3 promoter. The resulting constructs are pGL3-HtrA1-P3-mut1, mut2 and mut3.

The RXRα transcription factor binding sites were mutated based on a consensus nucleotide sequence in the HtrA1 P1 promoter. The resulting constructs are pGL3-HtrA1-P1-M1’, M2’, M3’, M4’, M5’ and M6’. M1’: − 732 ~ − 751, M2’: - 530 ~ − 546, M3’: − 425 ~ − 439, M4’: − 7 ~ + 16, M5’: both - 530 ~ − 546 and − 425 ~ − 439, M6’: − 732 ~ − 751, − 530 ~ − 546 and − 425 ~ − 439.

### HDAC activity assay

The in vitro HDAC activity assay for paired cell lines was performed with an HDAC fluorescent activity assay kit (BioVision, Milpitas, CA, USA) according to the manual in the kit.

### RXR activity assay

The in vitro RXR activity assay was performed on paired cell lines using an RXRα reporter assay kit (Cayman Chemical, USA) based on the manual in the kit.

### Chromatin immunoprecipitation (ChIP)

The interaction of the RXRα protein with the HtrA1 gene and influence of histone acetylation on HtrA1 promoter were analyzed using a ChIP kit (CST, Danvers, MA, USA) according to instruction manual provided by the manufacturer. Briefly, an RXRα antibody or H3ac/H4ac antibody were used for immunoprecipitation. HtrA1 promoter primers were used to carry out RT-PCR on DNA isolated from the ChIP experiment. After quantitative PCR, the amplification products were analyzed using agarose gel electrophoresis.

### mRNA microarray

Total RNA was extracted from H460, A549, H460/CDDP and A549/CDDP cells using a Trizol reagent (Thermo Fisher Scientific, Waltham, MA, USA). RNA sequencing analysis was performed to identify the differentially expressed genes between the parental cells and CDDP-resistant cells. Gene Set Enrichment Analysis (GSEA) was used for further analysis to identify the relevant genes.

### In vivo tumor growth model

NCI-H460 cells with a stable knockdown of HtrA1 were established by transfection with an sh-HtrA1 lentivirus. After confirmation by Western blot, 1 × 10^6^ sh-HtrA1 and control cells were injected into the right flank of 6- to 8-weekold male Balb/c-nu mice. Two weeks after the injections, mice were administered CDDP (5 mg/kg, once per week, intravenously) for three weeks. To establish the xenograft model of cisplatin-resistant lung cancer, 2 × 10^6^ A549/CDDP or H460/CDDP cells in 0.2 ml serum-free medium were injected into the right flank of 6- to 8-weekold male Balb/c-nu mice. When the average tumor volume reached 50–80 mm^3^, the mice were randomly divided into five treatment groups: control (saline only), CDDP (5 mg/kg, once per week, intravenously), DW22 (25 mg/kg, twice per week, intravenously), SAHA+bexarotene+CDDP (25 mg/kg, twice per week, 30 mg/kg, twice per week, 5 mg/kg, once per week, interval more than 2 h, intravenously) and DW22 + CDDP (25 mg/kg, twice per week, 5 mg/kg, once per week, interval more than 2 h, intravenously). The tumor size was measured once every 2 days using a caliper (tumor volume = 1/2 × shortest diameter^2^ × longest diameter). The body weight was also recorded once every 2 days. The mice were sacrificed after 14 days and the tumors were excised and stored at − 80 °C until analysis using the TUNEL assay and Western blotting. This protocol was approved by the Committee on the Ethics of Animal Experiments of the Shenyang Pharmaceutical University.

### Statistical analysis

Each experiment was repeated at least three times and results were shown as the mean ± SEM. The data were analyzed using Student’s t-test (Independent-Sample T Test) and a One-Way ANOVA analysis of variance (ANOVA), followed by post hoc analysis using Dunnett, using SPSS V 20.0 software (SPSS Inc., USA). The a priori significance level was *p* < 0.05.

## Supplementary information

**Additional file 1: Supplementary Figure 1.** The identification of HtrA1 as a cisplatin resistance-related gene in NSCLC. **A-B** The differential expression of genes in **A** A549 and A549/CDDP cells, **B** NCI-H1299 and NCI-H1299/CDDP cells by RT-PCR. **C** GSEA analysis of PPAR, RAR and acetyltransferase in A549/CDDP resistant cells. **D** The activity of HDAC and RXR in NCI-H1299/CDDP and NCI-H1299 cells. **E** The protein expression levels of the major isoforms of HDAC and RXR in NCI-H1299/CDDP and NCI-H1299 cells. **F** The protein expression of HtrA1 in CDDP resistant cells after SAHA and CG347B treatment. ^*^P < 0.05, **P < 0.01, ***P < 0.001, as compared to the parental group or ctrl group.

**Additional file 2: Supplementary Figure** 2 The downregulation of HtrA1 by HDAC and RXR increases the efficacy of cisplatin in NSCLC/CDDP resistant cells. **A** RT-PCR analysis of HtrA1 mRNA in NCI-H1299/CDDP cells transfected with HDAC1 or control siRNA. **B** RT-PCR analysis of HtrA1 mRNA levels in NCI-H1299/CDDP cells transfected with a RXRα overexpression or control plasmid. **C, E** RT-PCR analysis of HtrA1 in **C** CDDP resistant NSCLC cells and **E** parental NSCLC cells incubated with bexarotene, LBH-589 or bexarotene + LBH-589. **D** RT-PCR analysis of HtrA1 mRNA levels in parental NSCLC cells and CDDP resistant NSCLC cells incubated with bexarotene, SAHA or bexarotene + SAHA. **F-G** The protein expression of HtrA1 when silenced HDAC1, overexpressed RXRα and silenced HDAC1 and overexpressed RXRα simultaneously. **H** The mRNA expression of RXR isoforms when treated with SAHA and the mRNA expression of HDAC1 when treated with Bexa. **I** Cell migration assay in NCI-H1299 cells transfected with a HtrA1 or control siRNA. ^*^P < 0.05, ^**^P < 0.01, as compared to the control group or scram group or mock group.

**Additional file 3: Supplementary Figure 3** The prognosis of single HtrA1(A), or HtrA1 combined with HDAC1(B) in NSCLC cases from the ProgGENEV2 database.

**Additional file 4: Supplementary Figure 4**. Transcriptional activation of HtrA1 depends on RXRα heterodimeric complexes and HDAC activity. **A, B** Dual luciferase reporter assay for the transcriptional activity of four HtrA1 promoter fragments (P1-P4) in parental NSCLC cells. ^#^*P* < 0.05, ^##^*P* < 0.01, as compared to the pGL3-HtrA1-P1 construct. **C** The sequencing traces of mutated RXRα binding sites of HtrA1 promoter P1.. **D-E** Luciferase activity elicited by the HtrA1 P3 promotor in parental NSCLC cells after **D** overexpression of HDAC1 and **E** overexpression of RXRα. **F** The structure of DW22. **G** Luciferase activity elicited by the HtrA1 P3 promotor in parental NSCLC cells when treated with Bexa, SAHA and DW22. **H** The ChIP assay for the combination of HtrA1 and RXRα in NCI-H460/CDDP cells when incubated with DW22, bexarotene and SAHA. **I** Luciferase activity elicited by the HtrA1 P3 promotor in parental NSCLC cells incubated with different heterodimer activators. **J** RT-PCR analysis of HtrA1 mRNA levels in parental and CDDP resistant NSCLC cells incubated with different heterodimer activators. **K** The protein expression of HtrA1 in NCI-H1299/CDDP cells when incubated with different heterodimer activators. **L** Luciferase activity elicited by the HtrA1 promotor in NCI-H1299/CDDP cells incubated with bexarotene combined with different heterodimer activators. **M** The sequencing traces of mutated RXRα binding sites in the HtrA1 P3 promoter. The resulting constructs are pGL3-HtrA1-P3-mut1, mut2 and mut3. **N** Luciferase activity elicited by the HtrA1 P3 promotor constructs from **M**, with mutations in the RXRα binding sequences. ^&^*P* < 0.05, ^&&^*P* < 0.01, ^&&&^*P* < 0.001, as compared with pGL3-HtrA1-P3. **O** Luciferase activity elicited by the HtrA1 P3-mut2 promotor in parental NSCLC cells after incubation with SAHA, bexarotene, DW22 or SAHA + bexarotene. **P** The ChIP assay for the binding ability of promotor of HtrA1 with RXRα. ^#^P < 0.05, ^##^ P < 0.01, as compared to the P1 group or P3 + RXRα group. ^&^P < 0.05, as compared to the P3 + HDAC1 + RXRα group. ^*^P < 0.05, ^**^P < 0.01, ^***^P < 0.01, as compared with the control group.

**Additional file 5: Supplementary Figure** 5 The dual-target compound, DW22, significantly inhibits the growth of cisplatin-resistant cells by regulating HtrA1mRNA expression. **A** MTT assay indicating the sensitivity of parental NSCLC cells following incubation with DW22, cisplatin and DW22 + cisplatin for 72 h. DMSO was set as the control group that comparing to treatment groups. **B** The combination index for DW22 + cisplatin in parental NSCLC cell lines was calculated using the Calcusyn program. CI < 0.90 indicates synergism, 0.90–1.10 indicates an additive effect and > 1.10 indicates antagonism. **C** Migration and invasion assays in parental and CDDP resistant NSCLC cells. **D, E** The inhibitory efficacy of DW22, bexarotene, SAHA or bexarotene + SAHA on **D** invasion and **E** migration in CDDP resistant NSCLC cells. **F, G** The inhibitory efficacy of DW22, bexarotene, LBH-589 or bexarotene + LBH-589 on **F** invasion and **G** migration in A549/CDDP cells. **H-I** The mRNA and protein expression of HtrA1 after DW22 treatment. ^*^P < 0.05, ^**^P < 0.01, ^***^P < 0.001 as compared with VEGF group or control group. ^###^P < 0.001 as compared to the SAHA group. ^&^P < 0.05, ^&&&^P < 0.001 as compared to the bexarotene group.

**Additional file 6: Supplementary Figure 6** DW22 signficantly decreases cisplatin resistance in NSCLC by rescuing HtrA1 protein expression in vivo. **A, B** The effect of cisplatin, DW22 and cisplatin + DW22 on **A** tumor volume, tumor weight and **B** body weight in Balb/c-nu mice with A549/CDDP xenografts. **C** The effect of cisplatin, DW22, SAHA + bexarotene + CDDP and cisplatin + DW22 on the viscera index in Balb/c-nu mice with NCI-H460/CDDP xenografts. **D** The apoptosis proteins in A549/CDDP tumor tissues treated with CDDP, DW22 and CDDP + DW22. Four tumor tissues from four independent mice in each group were used for this analysis. Every two of them were mixed together randomly that shown as #1 and #2. **E** The protein expression level of HtrA1 and Acetylated Histone4 in NCI-H460/CDDP xenograft tumor tissues. ^*^P < 0.05, ^**^P < 0.01, ^***^P < 0.001, as compared to the control group.

**Additional file 7: Supplementary Table 1**. The IC_50_ values and RI values of NSCLC parental cells and CDDP resistant cells for 72 h. **Supplementary Table 2** Antibody information. **Supplementary Table 3** Primer Sequences

## Abbreviations

NSCLC: Non-small-cell lung carcinoma
CDDP: Cisplatin
HDAC: Histone deacetylases
RXR: Retinoid X receptor
RTCA: Real time cellular analysis
