# Supplementary figures and images for: Characterization of a novel HDAC/RXR/HtrA1 signaling axis as a novel target to overcome cisplatin resistance in human non-small cell lung cancer
Source: Mol Cancer. 2020 Sep 2;19:134. doi: 10.1186/s12943-020-01256-9 (PMC7466461; doi:10.1186/s12943-020-01256-9)

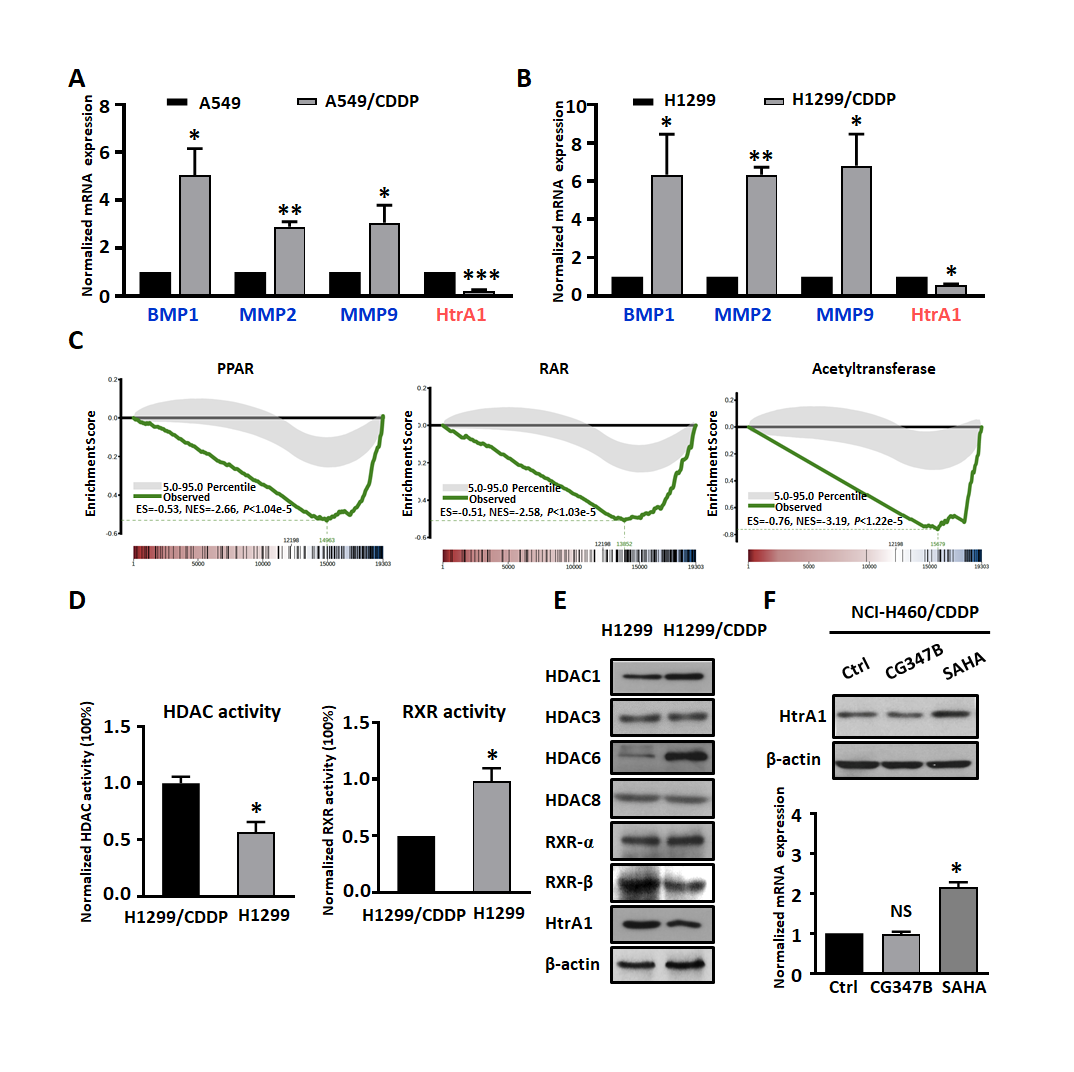

Supplement: Supplementary file 1 — Additional file 1: Supplementary Figure 1. The identification of HtrA1 as a cisplatin resistance-related gene in NSCLC. A-B The differential expression of genes in A A549 and A549/CDDP cells, B NCI-H1299 and NCI-H1299/CDDP cells by RT-PCR. C GSEA analysis of PPAR, RAR and acetyltransferase in A549/CDDP resistant cells. D The activity of HDAC and RXR in NCI-H1299/CDDP and NCI-H1299 cells. E The protein expression levels of the major isoforms of HDAC and RXR in NCI-H1299/CDDP and NCI-H1299 cells. F The protein expression of HtrA1 in CDDP resistant cells after SAHA and CG347B treatment. *P < 0.05, **P < 0.01, ***P < 0.001, as compared to the parental group or ctrl group. [file 12943_2020_1256_MOESM1_ESM.tif]

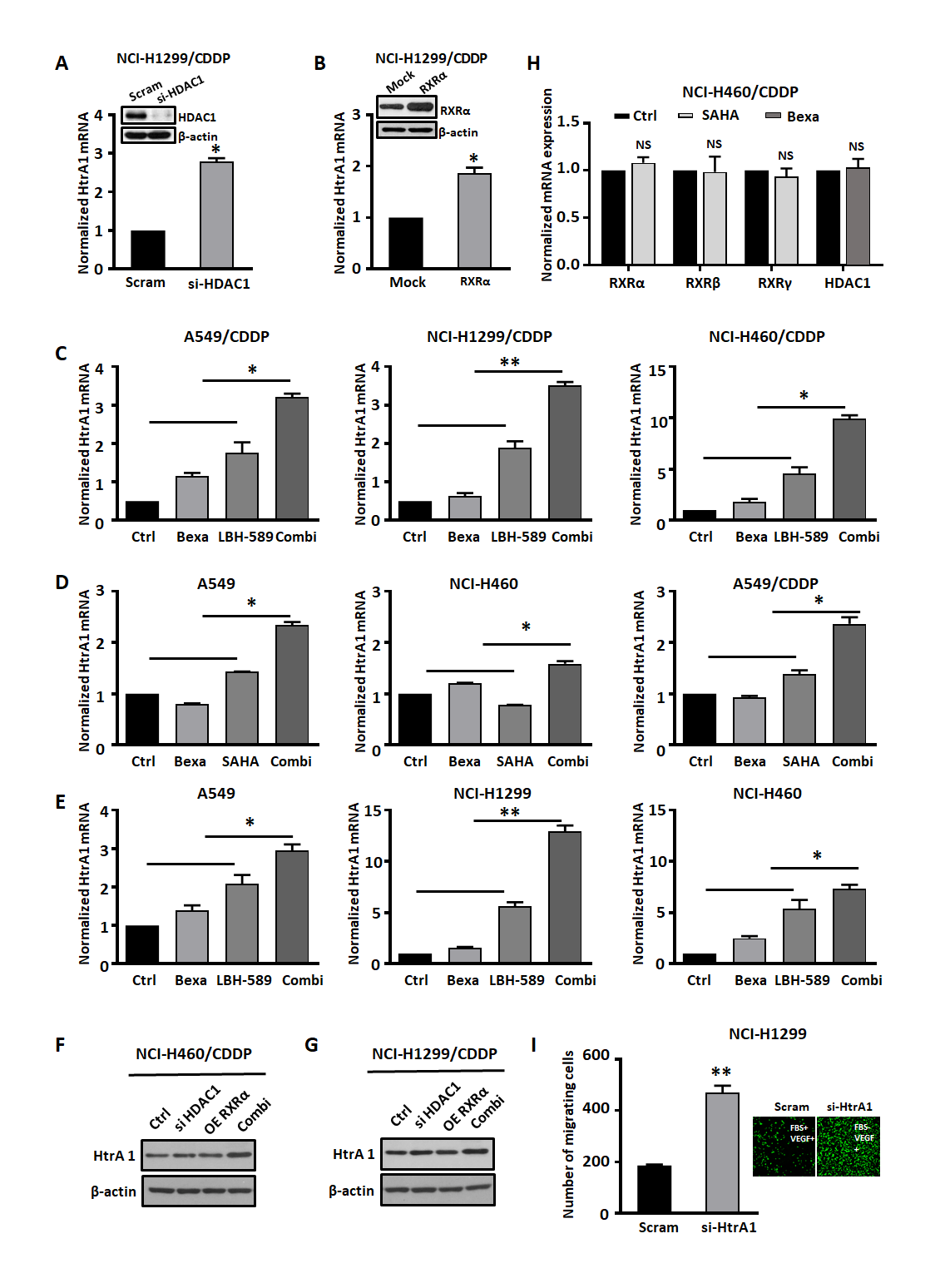

Supplement: Supplementary file 2 — Additional file 2: Supplementary Figure 2 The downregulation of HtrA1 by HDAC and RXR increases the efficacy of cisplatin in NSCLC/CDDP resistant cells. A RT-PCR analysis of HtrA1 mRNA in NCI-H1299/CDDP cells transfected with HDAC1 or control siRNA. B RT-PCR analysis of HtrA1 mRNA levels in NCI-H1299/CDDP cells transfected with a RXRα overexpression or control plasmid. C, E RT-PCR analysis of HtrA1 in C CDDP resistant NSCLC cells and E parental NSCLC cells incubated with bexarotene, LBH-589 or bexarotene + LBH-589. D RT-PCR analysis of HtrA1 mRNA levels in parental NSCLC cells and CDDP resistant NSCLC cells incubated with bexarotene, SAHA or bexarotene + SAHA. F-G The protein expression of HtrA1 when silenced HDAC1, overexpressed RXRα and silenced HDAC1 and overexpressed RXRα simultaneously. H The mRNA expression of RXR isoforms when treated with SAHA and the mRNA expression of HDAC1 when treated with Bexa. I Cell migration assay in NCI-H1299 cells transfected with a HtrA1 or control siRNA. *P < 0.05, **P < 0.01, as compared to the control group or scram group or mock group. [file 12943_2020_1256_MOESM2_ESM.tif]

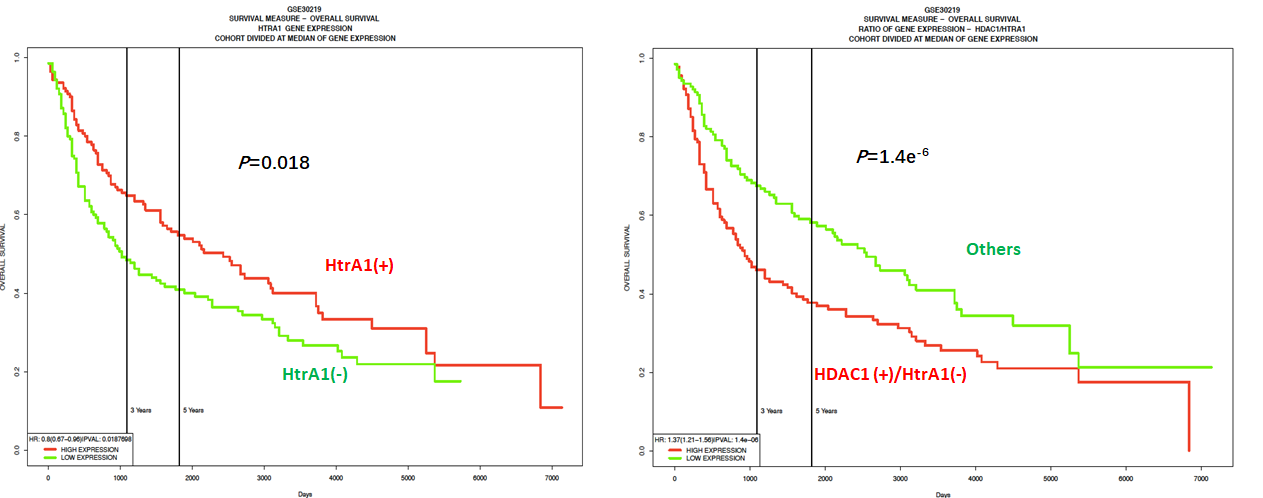

Supplement: Supplementary file 3 — Additional file 3: Supplementary Figure 3 The prognosis of single HtrA1(A), or HtrA1 combined with HDAC1(B) in NSCLC cases from the ProgGENEV2 database. [file 12943_2020_1256_MOESM3_ESM.tif]

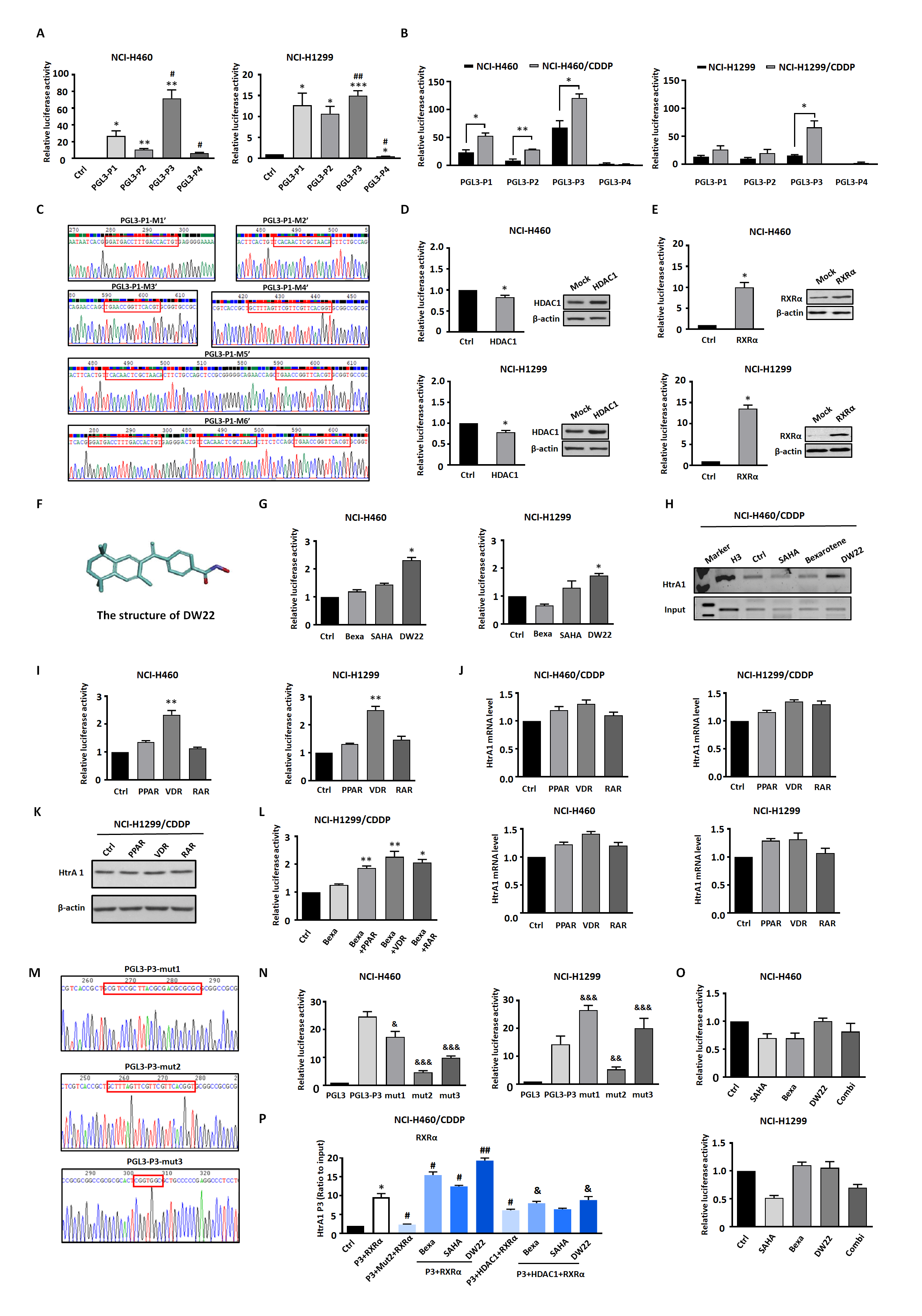

Supplement: Supplementary file 4 — Additional file 4: Supplementary Figure 4. Transcriptional activation of HtrA1 depends on RXRα heterodimeric complexes and HDAC activity. A, B Dual luciferase reporter assay for the transcriptional activity of four HtrA1 promoter fragments (P1-P4) in parental NSCLC cells. #P < 0.05, ##P < 0.01, as compared to the pGL3-HtrA1-P1 construct. C The sequencing traces of mutated RXRα binding sites of HtrA1 promoter P1.. D-E Luciferase activity elicited by the HtrA1 P3 promotor in parental NSCLC cells after D overexpression of HDAC1 and E overexpression of RXRα. F The structure of DW22. G Luciferase activity elicited by the HtrA1 P3 promotor in parental NSCLC cells when treated with Bexa, SAHA and DW22. H The ChIP assay for the combination of HtrA1 and RXRα in NCI-H460/CDDP cells when incubated with DW22, bexarotene and SAHA. I Luciferase activity elicited by the HtrA1 P3 promotor in parental NSCLC cells incubated with different heterodimer activators. J RT-PCR analysis of HtrA1 mRNA levels in parental and CDDP resistant NSCLC cells incubated with different heterodimer activators. K The protein expression of HtrA1 in NCI-H1299/CDDP cells when incubated with different heterodimer activators. L Luciferase activity elicited by the HtrA1 promotor in NCI-H1299/CDDP cells incubated with bexarotene combined with different heterodimer activators. M The sequencing traces of mutated RXRα binding sites in the HtrA1 P3 promoter. The resulting constructs are pGL3-HtrA1-P3-mut1, mut2 and mut3. N Luciferase activity elicited by the HtrA1 P3 promotor constructs from M, with mutations in the RXRα binding sequences. &P < 0.05, &&P < 0.01, &&&P < 0.001, as compared with pGL3-HtrA1-P3. O Luciferase activity elicited by the HtrA1 P3-mut2 promotor in parental NSCLC cells after incubation with SAHA, bexarotene, DW22 or SAHA + bexarotene. P The ChIP assay for the binding ability of promotor of HtrA1 with RXRα. #P < 0.05, ## P < 0.01, as compared to the P1 group or P3 + RXRα group. &P < 0.05, as co [file 12943_2020_1256_MOESM4_ESM.tif]

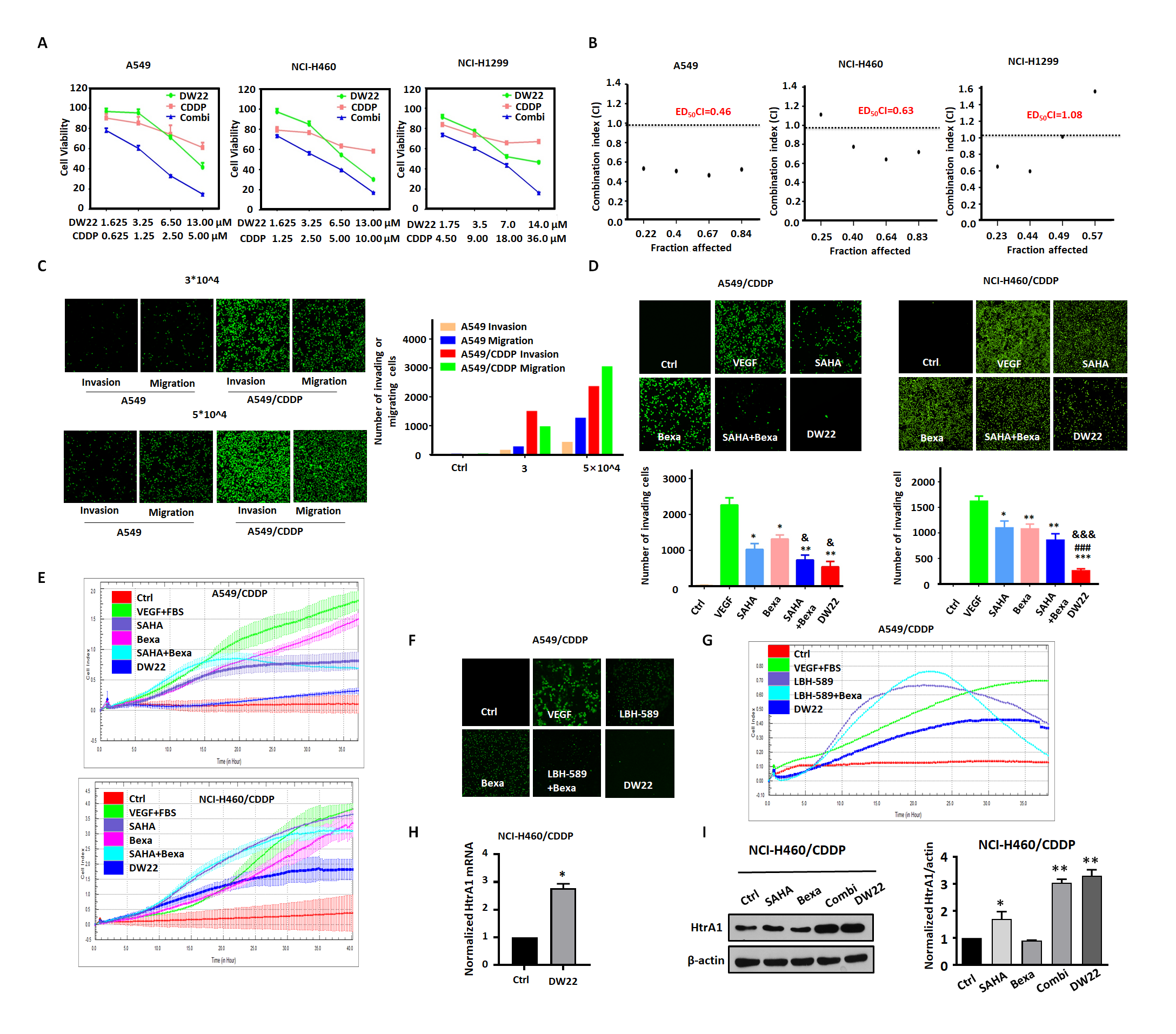

Supplement: Supplementary file 5 — Additional file 5: Supplementary Figure 5 The dual-target compound, DW22, significantly inhibits the growth of cisplatin-resistant cells by regulating HtrA1mRNA expression. A MTT assay indicating the sensitivity of parental NSCLC cells following incubation with DW22, cisplatin and DW22 + cisplatin for 72 h. DMSO was set as the control group that comparing to treatment groups. B The combination index for DW22 + cisplatin in parental NSCLC cell lines was calculated using the Calcusyn program. CI < 0.90 indicates synergism, 0.90–1.10 indicates an additive effect and > 1.10 indicates antagonism. C Migration and invasion assays in parental and CDDP resistant NSCLC cells. D, E The inhibitory efficacy of DW22, bexarotene, SAHA or bexarotene + SAHA on D invasion and E migration in CDDP resistant NSCLC cells. F, G The inhibitory efficacy of DW22, bexarotene, LBH-589 or bexarotene + LBH-589 on F invasion and G migration in A549/CDDP cells. H-I The mRNA and protein expression of HtrA1 after DW22 treatment. *P < 0.05, **P < 0.01, ***P < 0.001 as compared with VEGF group or control group. ###P < 0.001 as compared to the SAHA group. &P < 0.05, &&&P < 0.001 as compared to the bexarotene group. [file 12943_2020_1256_MOESM5_ESM.tif]

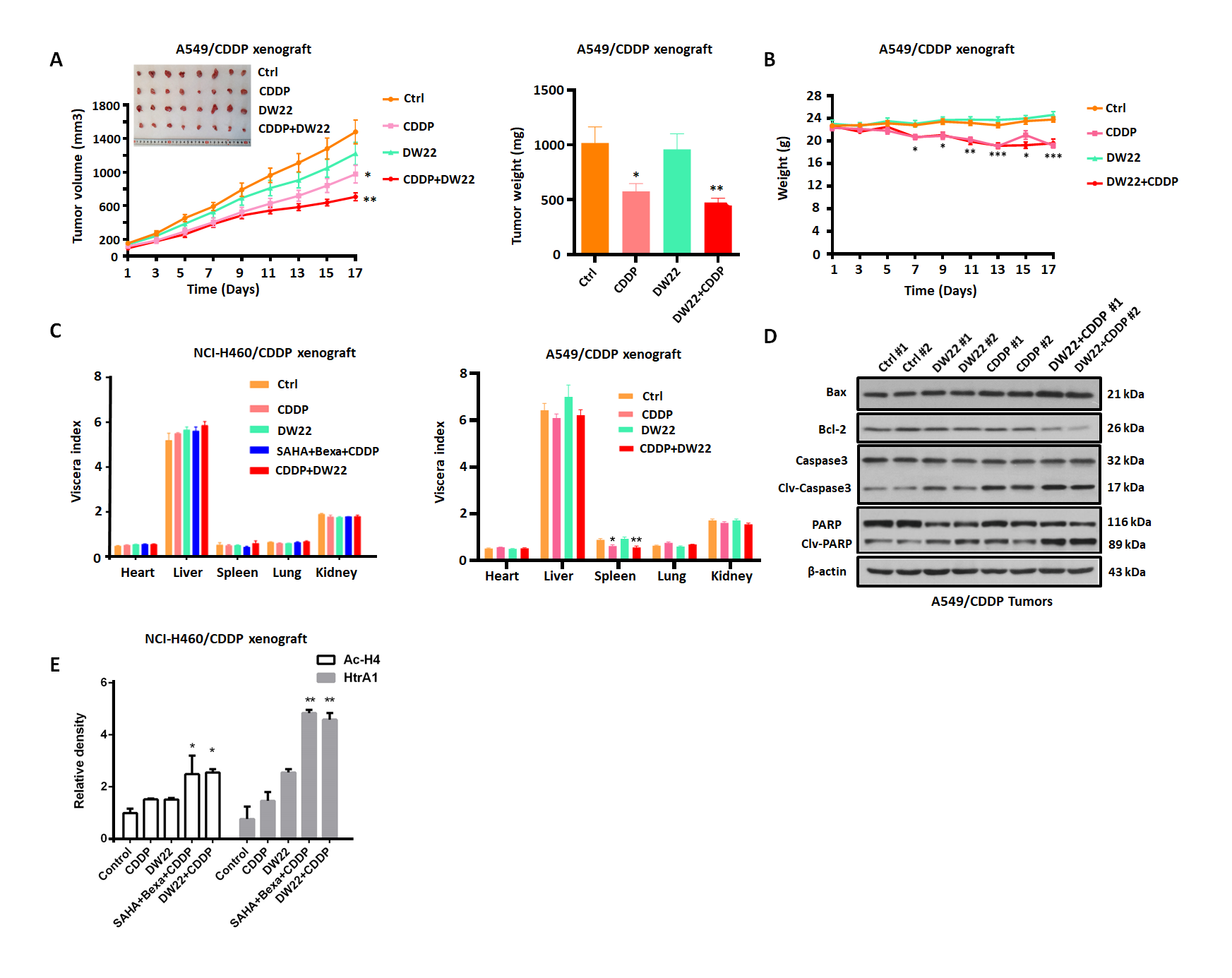

Supplement: Supplementary file 6 — Additional file 6: Supplementary Figure 6 DW22 signficantly decreases cisplatin resistance in NSCLC by rescuing HtrA1 protein expression in vivo. A, B The effect of cisplatin, DW22 and cisplatin + DW22 on A tumor volume, tumor weight and B body weight in Balb/c-nu mice with A549/CDDP xenografts. C The effect of cisplatin, DW22, SAHA + bexarotene + CDDP and cisplatin + DW22 on the viscera index in Balb/c-nu mice with NCI-H460/CDDP xenografts. D The apoptosis proteins in A549/CDDP tumor tissues treated with CDDP, DW22 and CDDP + DW22. Four tumor tissues from four independent mice in each group were used for this analysis. Every two of them were mixed together randomly that shown as #1 and #2. E The protein expression level of HtrA1 and Acetylated Histone4 in NCI-H460/CDDP xenograft tumor tissues. *P < 0.05, **P < 0.01, ***P < 0.001, as compared to the control group. [file 12943_2020_1256_MOESM6_ESM.tif]
