# Supplementary material for: Characterization of a novel HDAC/RXR/HtrA1 signaling axis as a novel target to overcome cisplatin resistance in human non-small cell lung cancer
Source: Mol Cancer. 2020 Sep 2;19:134. doi: 10.1186/s12943-020-01256-9 (PMC7466461; doi:10.1186/s12943-020-01256-9)
Supplement: Supplementary file 7 — Additional file 7: Supplementary Table 1. The IC50 values and RI values of NSCLC parental cells and CDDP resistant cells for 72 h. Supplementary Table 2 Antibody information. Supplementary Table 3 Primer Sequences [file 12943_2020_1256_MOESM7_ESM.docx]

Supplementary Table 1. The IC_50_ values and RI values of NSCLC parental cells and CDDP resistant cells for 72h

| Cell | CDDP/IC_50_(μM) | RI |
| --- | --- | --- |
| NCI-H1299 | 18.19±1.70 | 10.1 |
| NCI-H1299/CDDP | 184.55±2.87 |  |
| NCI-H460 | 4.98±0.35 | 11.3 |
| NCI-H460/CDDP | 56.05±1.92 |  |
| A549 | 2.23±0.14 | 9.6 |
| A549/CDDP | 21.46±2.13 |  |

Supplementary Table 2. Antibody information

| Antibody | Catalog | Company |
| --- | --- | --- |
| HDAC1 | 34589 | Cell Signaling Technology |
| HDAC3 | 10255-1-AP | Proteintech |
| HDAC6 | 7558 | Cell Signaling Technology |
| HDAC8 | 17548-1-AP | Proteintech |
| RXR-α | ab125001 | Abcam |
| RXR-β | 8715 | Cell Signaling Technology |
| HtrA1 | 55011-1-AP | Proteintech |
| RXRα-ChIP Grade | 3085 | Cell Signaling Technology |
| Ac-H3-ChIP Grade | ab47915 | Abcam |
| Ac-H4 | 06-866 | Millipore |
| Histone4 | 13919 | Cell Signaling Technology |
| GAPDH | 51332 | Cell Signaling Technology |
| α-tubulin | sc-5286 | Santa Cruz |
| β-actin | sc-8432 | Santa Cruz |

Supplementary Table 3. Primer Sequences

| Gene | Forward | Reverse |
| --- | --- | --- |
| HtrA1 | TTGTGTCGGAAGATGGACTG | GTGCGATGTCTGCTTTCTCA |
| β-actin | CTCCATCCTGGCCTCGCTGT | GCTGCTACCTTCACCGTTCC |
| BMP1 | ACAAGGACGAGTGCTCCAAG | TGTCACCTTGTGGTCACAGC |
| MMP2 | TGATCTTGACCAGAATACCATCGA | GGCTTGCGAGGGAAGAAGTT |
| MMP9 | CCTGGAGACCTGAGAACCAATC | CCACCCGAGTGTAACCATAGC |
| GAPDH | CAATGACCCCTTCATTGACC | GACAAGCTTCCCGTTCTCAG |
| HtrA1 promoter1 | CCTCTCGAGCTGATGACTCTCGGACTC | CCCAAGCTTATCTGCATGGCGACTCTG |
| HtrA1 promoter2 | CCGCTCGAGCGAATTACTTCTGCTCTC | CCCAAGCTTATCTGCATGGCGACTCTG |
| HtrA1 promoter3 | CTTCTCGAGCAGACGGGGAAACTGAGT | CCCAAGCTTATCTGCATGGCGACTCTG |
| HtrA1 promoter4 | AATTCTCGAGGAGGCCCTCCTGCACTCT | CCCAAGCTTATCTGCATGGCGACTCTG |
| RXRα | TCCTGTGACTGACTGTGA | GAACTGAATGGCGATGTATT |
| RXRβ | ACTCCTCATTGCCTCCTT | GCTCTGTCTTGTCCATCC |
| RXRγ | GGTGGAATGAATTGCTGATT | ATACTTCTGCTTGGTGTAGG |
| HDAC1 | GGAAATCTATCGCCCTCACA | AACAGGCCATCGAATACTGG |
| HtrA1-ChIP | AAACTGAGTCCCGCGAGAG | ATCTGCATGGCGACTCTG |
